# A panel of eight-miRNA signature as a potential biomarker for predicting survival in bladder cancer

**DOI:** 10.1186/s13046-015-0167-0

**Published:** 2015-05-21

**Authors:** Hui Zhou, Kun Tang, Haibing Xiao, Jin Zeng, Wei Guan, Xiaolin Guo, Hua Xu, Zhangqun Ye

**Affiliations:** Department of Urology, Tongji Hospital, Tongji Medical College, Huazhong University of Science and Technology, Wuhan, 430030 China; Institute of Urology, Tongji Hospital, Tongji Medical College, Huazhong University of Science and Technology, Wuhan, China

**Keywords:** Bladder cancer, miRNA profiles, miRNA signature, Prognosis, Integrated analysis

## Abstract

**Background:**

There is increasing evidence to suggest that miRNAs play an important role in predicting cancer survival. To identify a panel of miRNA signature that can divided tumor from normal bladder using miRNA expression levels, and to assess the prognostic value of this specific miRNA markers in bladder cancer (BCa).

**Methods:**

A comprehensive meta-review of published miRNA expression profiles that compared BCa and adjacent normal tissues was performed to determine candidate miRNAs as prognostic biomarkers for BCa. Vote-counting strategy and Robust Rank Aggregation method were used to identify significant meta-signature miRNAs.

**Results:**

We identified an eight-miRNA signature including three upregulated (miR-141, miR-200c, miR-21) and five downregulated (miR-145, miR-125, miR-199a, let-7c and miR-99a) miRNAs for the prediction of overall survival (OS) using TCGA dataset, and validated in our 48 BCa patients. X-tile plot was used to generate the optimum cut-off point and Kaplan-Meier method was used to calculate OS. A linear prognostic model of eight miRNAs was constructed and weighted by the importance scores from the supervised principal component method to divide patients into high- and low-risk groups. Patients assigned to the high-risk group were associated with poor OS compared with patients in the low-risk group (HR = 5.21, *p* < 0.001). Our validation cohort of 48 patients confirmed the panel of 8-miRNAs as a reliable prognostic tool for OS in patients with BCa (HR = 5.04, *p* < 0.001).

**Conclusion:**

The present meta-analysis identified eight highly significant and consistently dysregulated miRNAs from 19 datasets. We also constructed an eight-miRNA signature which provided predictive and prognostic value that complements traditional clinicopathological risk factors.

**Electronic supplementary material:**

The online version of this article (doi:10.1186/s13046-015-0167-0) contains supplementary material, which is available to authorized users.

## Background

Bladder cancer (BCa) is the top ten malignancy of all adult cancer and its incidence has increased over recent decades [1]. Current prognostic factors, namely tumor node metastasis (TNM) stage and pathological grade, are insufficient to predict individual clinical outcome [2]. These clinicopathological risk factors do not clearly distinguish between patients who have a high or low risk of disease recurrence, chemotherapy response and overall survival (OS). Thus there is a need to add new prognostic and predictive biomarkers to the current staging system, which also could be used as therapeutic targets.

MicroRNAs (miRNAs), a class of short, single-stranded and highly conserved noncoding RNA molecules, is recently discovered and shown to regulate gene expression at the post-transcriptional level, by binding through partial sequence homology, to the 3′-untranslated region (3′-UTR) of target mRNAs and causing translational inhibition or mRNA degradation. Recent evidence indicates a regulatory role for miRNAs in cancer development and progression, suggesting that miRNAs function as oncogenes or cancer suppressor genes in a tissue-specific manner [3]. MiRNAs also function in tumor cell proliferation, apoptosis, invasion and tumor vessel formation via the regulation of downstream targeted genes. Several studies showed that aberrant miRNA expression is related to overall survival, disease stage and the development of metastases and recurrences. Interestingly, recent studies proposed miRNA signature as promising biomarkers for early cancer detection and accurate prognosis as well as targets for more efficient treatment [4]. To date, only a few reports have described miRNA expression and its association with survival in bladder cancer.

MiRNA profiling datasets were emerging rapidly with the employ of high-throughput technologies. However, recent microarray datasets for miRNA showed inconsistent results between the studies due to different technological platforms and small sample size application. Considering the inconsistent annotation and ongoing discovery of new miRNAs, different detection methods used by different technological platforms and various methods for data processing and analysis, we are trying to find a meaningful way to increase the statistical power of microarray data by integrating the results of several individual studies. Hence, we performed an integrated miRNA expression profiling analysis to identify gene coexpression networks and to define more robust sets of cancer-related genes and miRNAs in BCa. The existing limitations on miRNA research prompted us to perform an integrated miRNA expression profiling analysis so as to (i) compare and validated the expression profiles of miRNA in BCa in comparison with NT, (ii) explore meta-signature miRNAs that consistently dysregulated expressed in BCa and their bioinformatics function, (iii) identify possible associations between miRNA expression patterns and OS in BCa, (iv) construct a prognostic model combing meta-signature miRNAs based panel and (v) provide a reliable prognostic and predictive biomarker that complements the traditional clinicopathological prognostic factors.

## Material and methods

### Selection of studies and datasets

#### Study selection

A systematic review of the literature was performed to identify miRNA expression profiling datasets comparing bladder cancer *vs*. normal tissue published up to Nov 2014. We conducted a systematic search of the electronic databases, including PubMed, GEO profile, TCGA and Oncomine dataset, using the MESH search headings: (mirna OR microrna OR mir-) AND profile AND (bladder urothelial) AND (cancer OR tumor OR carcinoma).

#### Inclusion criteria and exclusion criteria

To be included in the analysis, studies should meet the following criteria: (i), they were bladder cancer miRNA expression profiling studies; (ii), they used tissue samples obtained from surgically resected bladder cancer and corresponding adjacent or normal tissues for comparison; (iii), they reported the relative miRNAs expression using miRNA microarray, sequencing methods or directly qRT-PCR.

Studies were excluded in the meta-analysis if: (i) the inclusion criteria were not met, (ii) no outcomes of interest were reported or the necessary data were impossible to calculate or extrapolate from the published results, (iii) studies used the serum, plasma, exosome or urine samples of bladder cancer patients or cell lines, and (iv) the studies compared miRNA expression profiles in invasive and non-invasive bladder cancer.

#### Data extraction

Two reviewers (TK & ZH) extracted independently the following data including: first author, year of publication, country, study interval, assay type, No. of probes, cancer type, stage, No. of samples and the list of up- and down-regulated miRNA features, and their relative fold change. All disagreements about eligibility were resolved by discussion with a third reviewer (XH) until a consensus was reached.

### Bioinformatics analysis

#### Vote-counting rank

Vote-counting rank based of ranking potential molecular biomarkers was widely adopted in the meta-analysis [5]. The miRNAs were ranked according to their importance as follows: (i) number of comparisons consistently reported; (ii) total number of samples for comparison in agreement; (iii) average fold-changes reported for comparisons in agreement. Total sample size was considered more important than average fold-change because many studies did not report a fold-change.

#### Robust rank aggregation analysis

The list of extracted miRNAs was ranked based on their associated *p*-values (less than 0.05 was considered significant) when their fold-changes were not reported. All of the protocols for the Robust Rank Aggregation method are free to download at the comprehensive R Archive Network website (http://cran.r-project.org/). Details can be found in the package documentation (http://cran.r-project.org/web/packages/RobustRankAggreg/RobustRankAggreg.pdf). This method assigns a *p*-value to each element in the aggregated list, which indicates how much better it is ranked compared with a null model, expecting random ordering [6].

#### Cluster analysis of datasets

To assess the correlations between the results of individual datasets, hierarchical cluster analysis was used. Overall rank matrix was constructed based on rank matrixes obtained from separate analyses for upregulated and downregulated gene lists. In the matrix, value 0.5 means that this miRNA was not reported in that study, value above 0.5 means it is upregulated (one minus normalized rank of miRNA from the analysis of upregulated gene lists) and value below 0.5 means that this miRNA is downregulated in that study (normalized rank from analysis of downregulated gene lists). In cluster analysis, Spearman rank correlation with average linkage method was used.

#### miRNA target prediction

The putative targets of meta-signature miRNAs were predicted using databases utilizing three different target prediction algorithms. The mRNA targets of the miRNA genes were predicted using TargetScan (http://www.targetscan.org/), PicTar (http://pictar.mdc-berlin.de/), miRWalk (http://www.umm.uni-heidelberg.de/apps/zmf/mirwalk/), miRDB (http://mirdb.org/miRDB/), and miRANDA (http://www.microrna.org/microrna/get GeneForm.do), as each algorithm determines target binding differently. We selected targets with scores less than −1.25 for further analysis.

#### Enrichment analysis

A list of the potential targeted genes of each miRNA was submitted to DAVID Bioinformatics Resources 6.7 (http://david.abcc.ncifcrf.gov) to acquire the gene annotation, and a graph of signaling pathways for BCa carcinogenesis in which the target genes may be involved was generated using the KEGG pathways program (http://www.genome.jp/kegg/pathway.html). All statistical analyses were performed using Stata 10.0 software (StataCorp LP, College Station, TX) with two-tailed tests; statistical significance was defined as *p* < 0.05.

### Creation of a prognostic index model using the panel of 8-miRNAs signature based on TCGA dataset

#### Bladder cancer TCGA miRNA dataset

MiRNA expression data and corresponding clinical data for 84 bladder cancer patients were obtained from The Cancer Genome Atlas (TCGA) data portal [TCGA Data Portal.[https://tcga-data.nci.nih.gov/tcga/tcgaHome2.jsp]]. Both the miRNA expression data and clinical data, including outcome and staging information of TCGA patients deposited at the Data Coordinating Center (DCC), are publically available and open-access. TCGA data are classified by data type (clinical, mutations, gene expression) and data level, to allow structured access to this resource with appropriate patient privacy protection. This study meets the publication guidelines provided by TCGA [Publication Guidelines. [http://cancergenome.nih.gov/ publications/publicationguidelines]]. Normalized miRNA expression data were collected from the TCGA Data Portal using the Arraytool [7].

#### Prognostic value of each meta-signature miRNA in the TCGA dataset

SurvMicro (http://bioinformatica.mty.itesm.mx:8080/Biomatec/Survmicro.jsp) was used to investigate the relationship between each miRNA and overall survival within different independent classes of disease stage. The Kaplan-Meier and log-rank test were performed to test the equality for survival distributions in different groups. Hazard ratios (HRs), the ratio of hazards for a 2.5-fold change in the miRNA relative prognostic index, from survival analysis were used to identify candidate miRNAs associated with OS.

#### Prognostic index model creation using the 8-miRNAs signature based on multivariate Cox survival analysis

The TCGA validated method was used to develop a prognostic model of the weighted linear combination of the meta-signature miRNAs expression levels. This algorithm is based on an importance score assigned to each miRNA, calculated by the supervised principal components method. Using the Cox regression models, we calculated a risk score for each patient based on their individual expression levels of the eight miRNAs, where prognostic index (risk score) = (0.465 × expression value of miR-200c) - (0.607 × expression value of miR-141) - (0.294 × expression value of miR-21) + (0.085 × expression value of miR-99a) + (0.578 × expression value of miR-125b) - (0.218 × expression value of miR-199a) - (0.064 × expression value of miR-145) - (0.146 × expression value of let-7c).

### Validation of the 8-miRNAs signature expression using qRT-PCR

#### Sample collection

Forty-eight BCa and adjacent normal tissue samples (collected post-operatively from Dec 2010 to Dec 2014) used in this study were obtained from the Department of Urology in Tongji Hospital of Huazhong University of Science and Technology (Wuhan, China). The specimens were obtained from patients undergoing BCa resection with curative intent. All the diagnoses were based on pathological assessments. Upon removal of the surgical specimen, each sample was immediately frozen in liquid nitrogen and stored at −80 °C prior to RNA isolation and qRT-PCR analysis.

#### RNA extraction and qRT-PCR

Total RNA was isolated from the frozen tissue sample with TRIzol (Invitrogen) according to the manufacturer’s instructions. First-strand complementary DNA (cDNA) was synthesized from 2 μg of the total RNA using an oligo-dT primer and superscript II reverse transcriptase (Invitrogen). Then, quantification of the most up-regulated or down-regulated miRNAs was performed by qRT-PCR using SYBR Premix Ex Taq on MX3000 instrument. The U6 primers were obtained from GeneCopoeia. PCR was performed in a real-time PCR system as follows: 95 °C for 10 min, followed by 40 cycles of 95 °C for 10 s, 60 °C for 20 s and 72 °C for 30 s, and then 95 °C for 1 min and 60 °C for 1 min. All experiments were done in triplicate. The expression level values were normalized to those of the small nuclear RNA U6 as a control. Relative fold-changes of miRNA expression were calculated using the △△CT method, and the values were expressed as 2^-△△CT^.

### Validation of the 8-miRNAs signature for predicting survival in bladder cancer

#### Follow-up

The length of follow-up was calculated from the date of surgery to the date of last clinical follow-up. The spectrum of clinical follow-up included a history, physical examination, and routine biochemical examination.

#### Survival analysis

For survival analysis, we used the Kaplan-Meier method to analysis the correlation between recurrence-free survival, overall survival and the panel of 8-miRNAs signature, and the log-rank test was used to compare survival curves. We selected the optimum cut-off value for the 8-miRNAs signature using X-tile plots based on the association with the patients’ RFS and OS. X-tile plots provide a single and intuitive method to assess the association between variables and survival. The X-tile program can automatically select the optimum data cut point according to the highest χ^2^ value (minimum p value) defined by Kaplan-Meier survival analysis and log-rank test [8]. We did the X-tile plots using the X-tile software version 3.6.1 (Yale University School of Medicine, New Haven, CT, USA).

## Results

### Selection and a short overview of the datasets

A total of 19 bladder cancer (BCa) miRNA expression profiles were identified for this meta-analysis (Additional file 1: Figure S2). An expanded description of the design strategy is available in Additional file 2: Figure S1. First author, year of publication, acronym, country, period, cancer type, stage, sample size, microarray platform, No. of probes and cut-off criteria were extracted individually from each study and listed in Additional file 3: Table S1. A short description of the studies and the acronyms by which the studies are referred to in the following text is also provided in Additional file 3: Table S1.

798 different expressed miRNAs were reported in the 19 miRNA expression profiling datasets; 54 were up-regulated (Additional file 4: Table S2) and 49 were down-regulated (Additional file 5: Table S3) in at least three datasets. Although there were differences between the individual miRNA profiling datasets, the top lists of the deregulated miRNAs remained consistent from study to study.

**Fig. 1:**
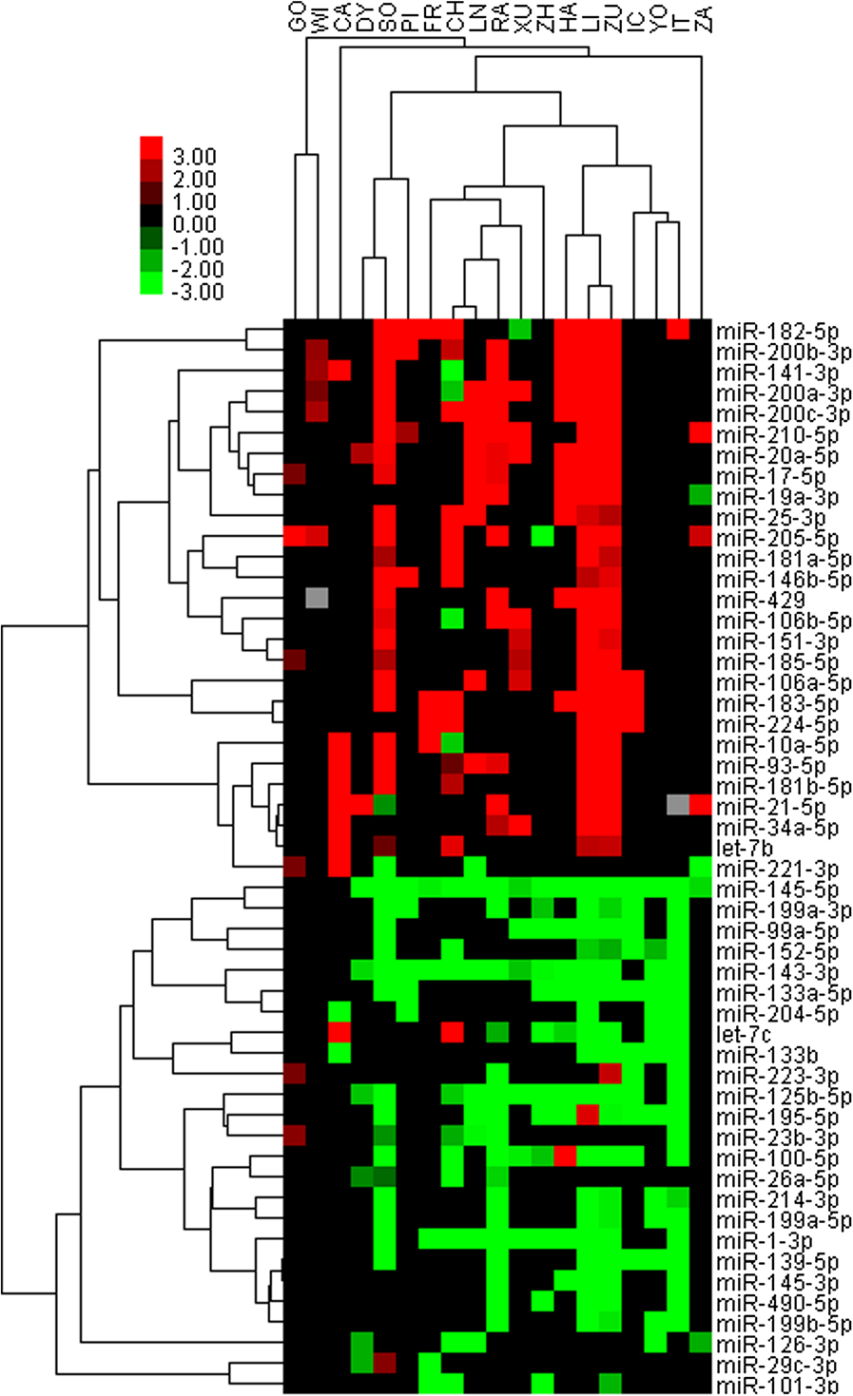
Heat map shows relative fold change of miRNAs in bladder cancer compared with normal adjacent tissue as reported by eligible studies. Hierarchical clustering of 19 selected studies and datasets with the 49 deferentially expressed miRNAs using average linkage clustering. Here we selected 52 miRNAs (26 down-regulated miRNAs and 26 up-regulated miRNAs) which reported in at least five expression profiling studies. Every row represents an individual miRNA, and each column represents an individual dataset. Acronyms are explained in Additional file 3: Table S1 and the number of miRNAs analyzed in each study is graphically depicted on the right. Pseudocolours indicate transcript levels from low to high on a log 2 scale from −3 to 3, ranging from a low association strength (dark, black) to high (bright, red, or green). Short red and green vertical bars indicate upregulated and downregulated miRNAs, respectively. While the black bar with the pseudocolours 0 means that there is no available data reported in the primary studies

To display the concordance of differentially miRNA profiling datasets more direct-viewing, heat-map and hierarchical clustering analysis were performed (Fig. 1). It shows that the results of the studies tend to cluster mainly according to utilized profiling platform. It also clearly shows that most of the miRNA are consistently upregulated and downregulated. The eight meta-signature miRNAs are the frenquently reported, whereas only one study CH reported a reversed result of miR-141 expression in BCa [9].

### Identification of the eight-miRNAs signature

We identified a statistically significant meta-signature of three upregulated and five downregulated miRNAs using robust rank aggregation (Table 1). Eight of the most significantly dysregulated miRNAs, miR-141, miR-200c, miR-21, miR-145, miR-125b, miR-199a, let-7c and miR-99a are reported in the majority of the datasets (8, 8, 6, 16, 12, 10, 9 and 8, respectively, Table 2). The direction of expression change of meta-signature miRNAs is consistent across all studies. The two meta-signature upregulated miRNAs (miR-141, miR-200c) belong to the cluster of miR-200 family which plays an important in tumor epithelial-mesenchymal transition (EMT). Our results from the vote-counting strategy were almost the same with those from the Robust Rank Aggregation method.

**Table 1:** BCa meta-signature miRNAs

miRNA name	Corrected p-value	Permutation p-value	No. of studies
Up-regulated			
hsa-miR-141-3p	5.51E-11	6.47E-13	8
hsa-miR-200c-3p	4.62E-10	5.33E-12	8
hsa-miR-21-5p	2.58E-9	4.42E-11	6
Down-regulated			
hsa-miR-145-5p	3.46E-11	4.25E-13	16
hsa-miR-125b-5p	6.71E-11	7.94E-13	12
hsa-miR-199a-5p	5.44E-10	6.38E-12	10
hsa-let-7c	2.31E-09	4.18E-11	9
hsa-miR-99a-5p	8.78E-08	9.32E-10	8

**Table 2:** Ten GO processes and pathways most strongly enriched by meta-signature miRNA targets

GO processes	Process	*p*-Value	Benjamini	Genes
	0035556: intracellular signaling cascade	7.8E-13	3.3E-9	258
	0007167: enzyme linked receptor protein signaling pathway	9.3E-10	2.0E-6	89
	0006796: phosphate metabolic process	1.1E-8	1.1E-5	194
	0007169: transmembrane receptor protein tyrosine kinase signaling pathway	9.6E-8	8.2E-5	61
	0035556: protein kinase cascade	1.1E-7	7.8E-5	88
	0006468: protein amino acid phosphorylation	2.2E-7	1.2E-4	138
	0006355: regulation of transcription	2.4E-7	1.1E-4	435
	0019220: regulation of phosphate metabolic process	2.5E-7	9.5E-5	107
	0031328: positive regulation of cellular biosynthetic process	1.7E-6	3.7E-4	137
	0043549: positive regulation of cell proliferation	2.6E-6	4.7E-4	91
KEGG Pathways	Pathway	*p* -Value	Benjamini	Genes
	04722: Neurotrophin signaling pathway	5.0E-12	9.1E-10	49
	04012: ErbB signaling pathway	8.2E-8	7.5E-6	33
	05220: Chronic myeloid leukemia	9.5E-8	5.7E-6	30
	05200: Pathways in cancer	1.7E-7	7.6E-6	81
	04520: Adherens junction	1.9E-7	6.8E-6	30
	04910: Insulin signaling pathway	1.6E-6	4.3E-5	41
	05215: Prostate cancer	1.8E-6	4.1E-5	31
	04722: p53 signaling pathway	8.0E-6	1.6E-4	25
	04010: MAPK signaling pathway	1.2E-5	2.2E-4	64
	05211: Renal cell carcinoma	1.4E-5	2.3E-4	25
Panther pathways	Pathway	*p* -Value	Benjamini	Genes
	P00018: EGF receptor signaling pathway	6.3E-6	7.9E-4	43
	P00047: PDGF signaling pathway	1.9E-4	8.1E-3	47
	P04398: p53 pathway feedback loops 2	2.0E-4	6.4E-3	22
	P00059: p53 pathway	3.5E-4	8.8E-3	34
	P00021: FGF signaling pathway	6.8E-4	1.4E-2	36
	P04393: Ras Pathway	3.5E-3	6.1E-2	25
	P04397: p53 pathway by glucose deprivation	6.4E-3	9.6E-2	11
	P00042: Muscarinic acetylcholine receptor 1 and 3 signaling pathway	8.3E-3	1.1E-1	18
	P00039: Metabotropic glutamate receptor group III pathway	1.2E-2	1.4E-1	21
	P00034: Integrin signalling pathway	1.6E-2	1.2E-1	47

The number of predicted target genes in the process or pathway is shown

A total of 8676 genes were identified as potentially targets of miRNAs contained in the eight-miRNA signature. We used six up-to-date prediction algorithms (TargetScan, PicTar, miRanda, miRWalk, miRDB and DIANA) to identify conservative target genes that could be predicted by the selected eight meta-signature miRNAs. We observed an overlap of targeted genes enriched in the eight meta-signature miRNAs. Geneset enrichment analysis found that meta-signature miRNAs cooperatively target functionally related and biologically relevant genes in signaling and developmental pathways. The top ten GO processes and pathways that were most strongly enriched with respect to the meta-signature miRNA candidates are shown in Table 2. This analysis revealed an overrepresentation of the predicted miRNA targets involved in the critical pathway linked to carcinogenesis such as: intracellular signaling cascade, ErbB signaling pathway, pathways in cancer, p53 signaling pathway, MAPK signaling pathway, and renal cell carcinoma. These indicate a potentially important functional role of selected meta-signature miRNAs in the progression of bladder cancer. Taken together, these exploratory analyses suggested that deregulation of the eight miRNAs might affect the critical pathways involved in cancer progression. We also had a review of the identified eight meta-signature miRNAs and their involvement in cancer pathogenesis in bladder cancer (Additional file 6: Table S4).

### Creation of the eight-miRNAs signature and its association with BCa overall survival in TCGA dataset

**Fig. 2:**
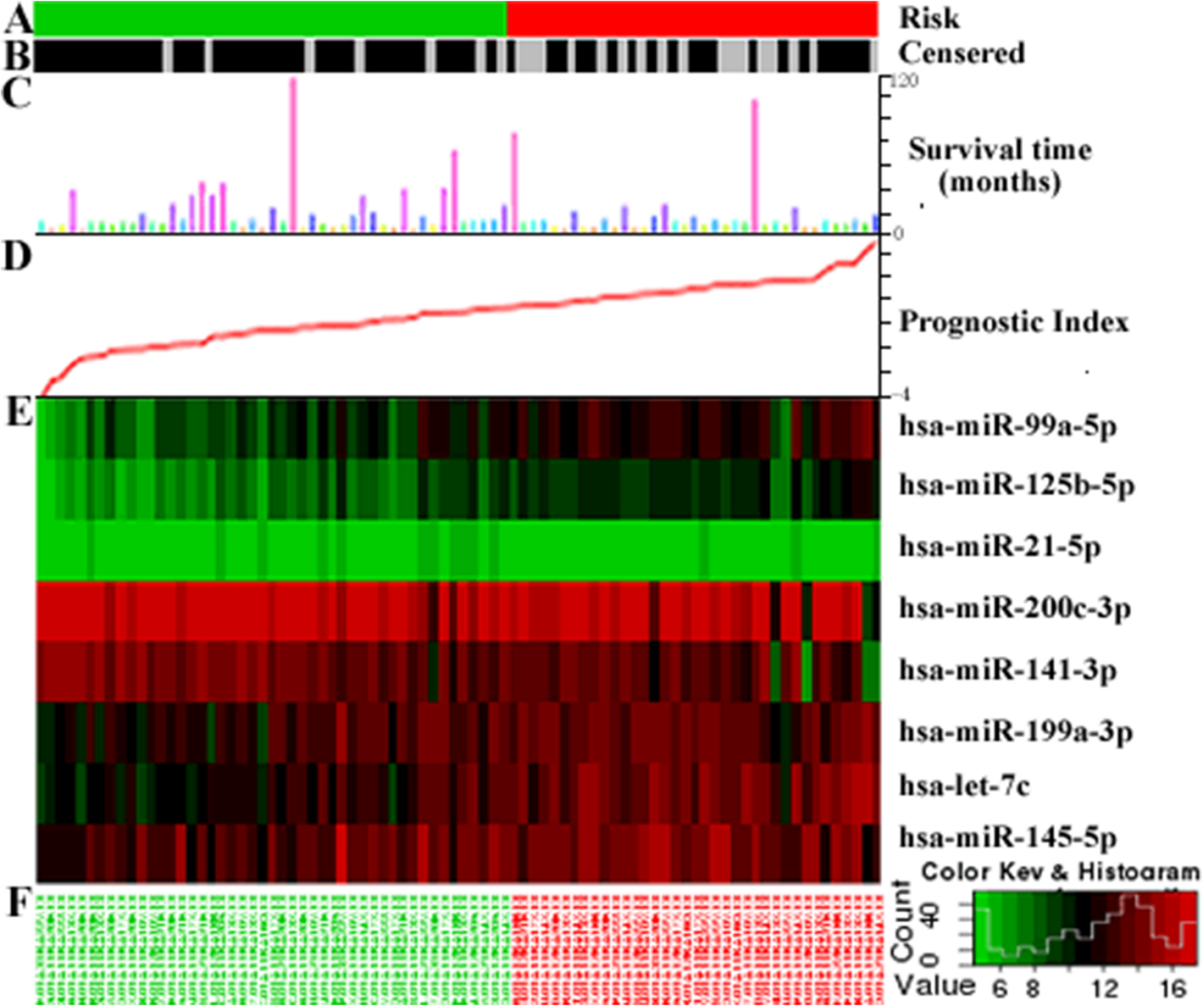
miRNA predictor-score analysis of 84 patients in TCGA cohort. Information related to censoring event being analyzed (risk group assignment (**a**), censoring status (**b**), time related to event (**c**), and prognostic index (**d**). Color-gram of miRNA expression profiles of TCGA patients. miRNA expression profiles shown as a heatmap helping in the analysis and visual correlation of the survival analysis and gene expression. Samples are shown in x-axis while genes are shown in y-axis (**e**). The genes are clustered by Euclidean distance. The patients’ ID showed under the figure (**f**)

**Fig. 3:**
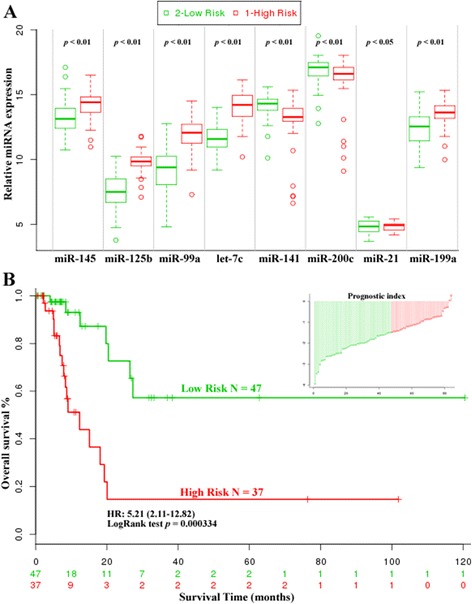
The eight-miRNA signature was tightly associated with prognosis in TCGA dataset. Box plot visualizing the expression levels of each miRNA in the risk groups generated (**a**). The cutoff value divided patients into low-risk and high-risk groups according to miRNA signature expression, and risk group splitting was optimized using a simple algorithm shown in the section Risk Groups Plots using the inner-group p-value. Kaplan-Meier overall survival analysis showed the eight-miRNA signature could predict the clinical outcome of TCGA (**b**)

We identified eight dysregulated meta-signature miRNAs in BCa, and bioinformatics analysis revealed important biological function in BCa progression. Then we tried to evaluate the prognostic power of the eight-signature miRNAs using the TCGA dataset of bladder cancer. Fig. 2 shows the distribution of patient prognostic scores, the survival status and tumor miRNA expression of all 84 TCGA BCa patients, ranked according to the prognostic score values for the eight-miRNA signature. Of all these eight miRNAs, six were associated with high risk (miR-21, miR-145, miR-125b, miR-199a, miR-99a, and let-7c) and two were shown to be protective (miR-200c and miR-141) (Fig. 3a). BCa with high prognostic scores tended to express high-risk miRNAs, whereas BCa with low prognostic scores tended to express protective miRNAs (Fig. 3d and e). Patients with high-risk prognostic scores had more chance of death than low-risk-score patients.

The max risk cutoff point was used for the TCGA patient cohort to classify the patients into either high-risk or low-risk groups. We then derived a formula to calculate the prognostic index (risk score), weighted by regression coincident based on multivariate Cox analysis of the 8-singature miRNAs. Patients expressing the high-risk miRNA signature exhibited poorer OS than patients expressing the low-risk miRNA signature (HR = 5.21, *p* < 0.001). Kaplan-Meier curves for the high-risk and low-risk groups within the TCGA cohort (n = 84) are shown in Fig. 3b. Time-dependent ROC curves were used to assess the prognostic power of the eight-miRNA signature. The area under curve (AUC) for the eight-miRNA signature prognostic model was 0.74 (*p* < 0.01, Additional file 7: Figure S12). We also elevated the predictive value of single miRNA, all of which were significantly associated with overall BCa survival in the TCGA dataset (*p* < 0.01, Additional file 8: Figure S3, Additional file 9: Figure S4, Additional file 10: Figure S5, Additional file 11: Figure S6, Additional file 12: Figure S7, Additional file 13: Figure S8, Additional file 14: Figure S9, Additional file 15: Figure S10). A list of survival analysis using TCGA datasets upon relative miRNAs that refered in at least three datasets were showed in Additional file 16: Figure S11 and Additional file 17: Table S5.

### Experimental validation of the relative expression levels of the eight-miRNAs in patients with bladder cancer

Considering of the basis of the miRNA microarray results, we further examined BCa-associated miRNAs expression using qRT-PCR to analysis the 48 BCa samples in different sets so as to assess and validate the prognostic value of candidate miRNAs. Our cohort of bladder cancer patients’ clinicopathological characters was listed in Additional file 18: Table S6. We selected the panel of eight meta-signature miRNAs for the validated qRT-PCR analysis. The results showed that the expression levels of miR-21, miR-141 and miR-200c were increased, whereas the levels of miR-145, miR-125b, miR-199a, miR-99a and let-7c were decreased in the BCa tissues compared with adjacent normal tissue (all *p* < 0.01). Detailed data are available in Additional file 19: Figure S13.

**Fig. 4:**
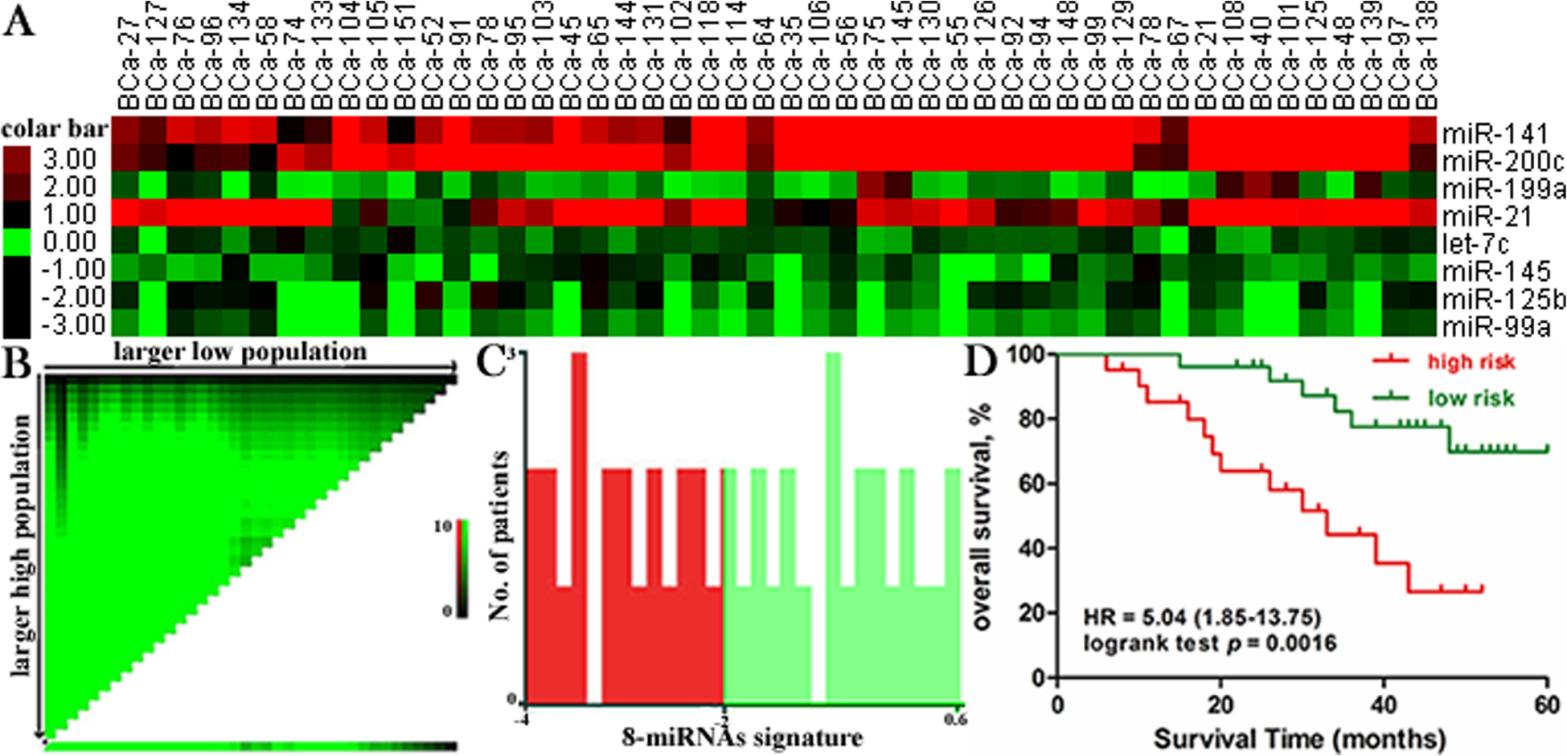
Expression and Kaplan-Meier overall survival analysis of the eight-miRNAs signature in our validation cohort of 48 bladder cancer patients. Heat map shows relative fold change of miRNAs in bladder cancer compared with normal adjacent tissue determined by qRT-PCR (**a**). Hierarchical clustering of 48 paired tumor tissues and adjacent normal tissue with the 8 deferentially expressed miRNAs using average linkage clustering. Every row represents an individual miRNA, and each column represents an individual sample. Pseudocolours indicate transcript levels from low to high on a log 2 scale from −3 to 3, ranging from a low association strength (dark, black) to high (bright, red, or green). Kaplan-Meier recurrence-free survival and overall survival analysis by X-tile plots cut-off point (**b**, **c**, **d**). X-tile plots of training sets are shown in the left panels. The plot showed the chi-squared log-rank values created when the cohort was divided into two groups. The optimal cut-point highlighted by the black circle in the left panels (**b**) is shown on a histogram of the entire cohort (middle panels, **c**) and a Kaplan-Meier plot (right panels, **d**). P value was determined by using the cut-point defined in the training subset to parse a separate validation subset. The optimal cut-point for prognostic index determined by X-tile analysis from the validation cohort and reached high statistical significance

To display the relative expression of differentially miRNA more direct-viewing, heat-map and hierarchical clustering analysis were also performed (Fig. 4). Short red and green vertical bars indicate upregulated and downregulated miRNAs, respectively. Using hierarchical clustering, based on the differentially expressed miRNAs, we could successfully separate the 8 miRNAs into two discrete groups, three upregulated miRNAs and five downregulated miRNAs (Fig. 4a).

### Validation of the 8-miRNAs signature for predicting survival in bladder cancer

The same prognostic score formula obtained from the TCGA dataset was used to calculate the eight-miRNA signature score for each of our 48 patients in the validation cohort. We used X-tile plots to generate the optimum cut-off point for the eight-signature miRNAs in our validation cohort (Fig. 4b, c, d). Cox regression analysis validated the panel of eight-miRNA signature as a potential prognostic biomarker (HR: 5.04, 95%CI: 1.85-13.75, *p* = 0.0016).

## Discussion

Recent studies indicated miRNAs played an important role in predicting OS in BCa. Here, we performed an integrated analysis approach to analyze BCa specific miRNAs derived from independent profiling datasets. We used robust rank aggregation method and identified a meta-signature of three upregulated and five downregulated miRNAs. Deriving from TCGA dataset, we identified eight-miRNA signature and constructed a prognostic model closely associated with both biological function and clinical survival. Our validation also highlighted the potential of miRNA panel to facilitate clinical prognosis prediction in patients with BCa.

### We compared and validated the expression miRNA in BCa with NT

Our cluster-analysis distinguished the significantly upregulated and downregulated miRNAs in BCa comparing to NT, however, recent miRNA expression profiling datasets are lack of inconsistent results between the studies due to different technological platforms and lab protocols as well as small sample sizes application. This inconsistence may be also caused, at least in part, by fundamental, methodological differences in the preselection of candidate miRNAs. Although the preferred method for miRNA expression meta-analysis involves analysis of the raw expression datasets that are pooled together, such rigorous approach is often not possible due to the unavailability of the raw data. Variations in the number of miRNAs known at the moment and the technological platform employed in any particular study make the proper integration of raw datasets very complicated. In addition, the relatively small sample size and noisiness of microarray data have resulted in inconsistency of biological conclusions.

For example, Liu et al. reported that miR-200c was down-regulated in bladder cancer tissues and cell lines [10]. According to our integrated analysis based 19 miRNA profiling datasets and validation, miR-200 family were up-regulated in BCa compared with normal tissue. However, as we all knew miR-200c was tightly associated with EMT, and up-regulated miR-200c could inhibit proliferation, invasion and migration in cancer progression. It seemed contradictory between miR-200c relative expression and its biological function. However, we found miR-200c was downregulated in invasive comparing non-invasive BCa. Therefore, we could still say up-regulated miR-200 family inhibit EMT and provide favorable prognosis in BCa. So, the authors might reverse miRNA expression just to meet expectation of its tumor function. Here, we confirmed that miR-200c was upregulated in BCa vs. NT, and downregulated in invasive vs. noninvasive BCa, also miR-200c was downregulated in BCa urine vs. normal urine [11], while circulating miR-200b was upregulated in plasma [12] and serum [13]. Also, miR-199a-3p is highly expressed in hair follicles and in some tumor cells, suggesting its participation in tumor progression, but it is significantly downregulated in hepatocellular carcinoma and in bladder cancer.

### We identified an eight-miRNA signature closely associated with both biological function and clinical survival

In this study, we selected common miRNAs related to clinical outcome as the potential prognostic miRNAs. Among the eight miRNAs, miR-21 has been validated as a prognostic and predicative biomarker in various cancers [14]. miR-21 is shown to significantly upregulated in tumor tissues and may modulates cell proliferation and sensitivity to doxorubin [15]. MiR-21 is found to be significantly upregulated in high-grade tumors in contrast to low-grade ones [16], also it may serve as independent prognostic factors for overall survival and recurrence [17], thus it has the prospect of becoming candidate biomarker for BCa.

MiR-200 family appears to control the EMT process and sensitivity to EGFR therapy in bladder cancer cells [18]. MiR-141 is upregulated in bladder cancer compared to NT. However, increased expression of miR-141 is independently associated with favorable prognosis of bladder cancer patients, thus it may serve as a promising biological marker for diagnosis and risk evaluation of bladder cancer. However, no study had reported the function of miR-200b in BCa, which need to be further investigated.

MiR-125b was found to regulate G1/S transition through the E2F3-Cyclin A2 signaling pathway [19]. Recent findings show that upregulation of miR-125b inhibited proliferation, motility and increased apoptosis by suppressing SIRT7 and MALAT1 [20]. It can also suppresses cell migration and invasion by directly targeting matrix metalloproteinase 13 (MMP13) in bladder cancer and may be promising molecular marker and therapeutic target to inhibit bladder cancer metastasis [21]. Taken together, these studies indicate that miR-125b may act as a tumor suppressor in bladder cancer and downregulation of miR-125b may contribute to the tumorigenesis and development.

MiR-143/-145 cluster located at chromosome 5q33, a well-known fragile site in human genome. As expected, we found that these two miRNAs were highly co-downregulation in bladder cancer. MiR-143 and miR-145 have well-documented antiproliferative and proapoptotic effects due to their ability to downregulate a host of target genes, including plasminogen activator inhibitor-1 (PAI-1) [22], FSCN1 [23], PAK1 [24] and insulin-like growth factor receptor I (IGF-IR) [25]. These two miRNAs have also been reported to be implicated in Kras regulation and apoptosis avoidance. Moreover, by targeting socs7, exogenous miR-145 can promote the nuclear translocation of STAT3, which regulates the IFN-β induction [26]. Several studies have also demonstrated that miR-143 and miR-145 inhibit multiple tumor survival effectors, thus exerting antitumor effect in cancer therapy. For instance, via regulating PI3K/Akt and MAPK signaling pathways, the growth of bladder cancer cells is attenuated after replacement treatment with miRNA-143 and −145 [27].

Down-regulated miRNA-99a can act as a tumor suppressor by inhibiting cell proliferation, migration and invasion by targeting fibroblast growth factor receptor 3 (FGFR3) in bladder cancer [28]. Furthermore, the expression of miR-99a was investigated and shown to be decreased in plasma of bladder cancer patients, which strongly supported miR-99a as the potential diagnostic marker of bladder cancer.

For the remaining two miRNAs (let-7c and miR-199a-5p), to our knowledge, are less studied in bladder cancer. MiR-199a-5p is downregulated in bladder cancer and seems to suppress chemoresistance by targeting the endoplasmic reticulum chaperone and signaling regulator GRP78 [29]. Let-7c might play a role in the bladder cancer pathway along with other miRNA and genes [30].

Our gene ontology and pathway enrichment analysis *in silico* based on the putative targeted genes, suggested that variation in miRNAs expression might affect critical pathways involved in BCa progression. A better understanding of the targeted genes of the miRNAs would advance their use in clinical settings. As shown in Additional file 6: Table S4, dysregulated miRNAs in RCC involved in cancer pathogenesis in other malignancies with universal biological function and targeted genes. Since target prediction algorithms generate certain false negatives and false positives, further investigations is warranted in our sequent study.

### We constructed a reliable prognostic model based miRNA expression using TCGA dataset and our validation cohort

Because of the lack of cancer specificity and sensitivity, single miRNA has limitation in the prognostic and predicative of BCa. Recently, many studies emerging identified the important role of special miRNA signature in predicating cancer progression [31, 32]. Liu et al. identified a five-miRNA signature which might add prognostic value to the TNM staging system and optimize treatment decisions for patients at high risk of progression in nasopharyngeal carcinoma [4]. In another study, Chen et al. analyzed the miRNA expression profile of 119 paired ESCC samples by microarray and identified a four-miRNA signature that predicted patient survival [33]. Zhang et al. identified a six-miRNA-based classifier as a reliable prognostic and predictive tool for disease recurrence in patients with stage II colon cancer, and it might be able to predict which patients might benefit from adjuvant chemotherapy [34]. Since we considered single miRNA had limit in predicting BCa survival, therefore, we built a classifier based on the eight miRNAs: miR-21, miR-200c, miR-141, miR-145, miR-125b, miR-199a, let-7c and miR-99a.

### Advantage and limitation

Up to date, no integrated analysis of miRNA profiling studies has investigated in BCa specially. The present meta-analysis has been proved to be useful in exploring candidate miRNA biomarkers in human BCa. The present study suggested eight promising miRNAs that have been consistently reported with fold change. Their potential targets may provide some clues of miRNAs in tumorigenesis and the underlying mechanisms.

However, we should admit that there exist certain limitations in our integrated network analysis. The major limitation of this study is the inconsistently results of eligible miRNA expression profiling datasets detected with different technological platforms. Second, the sample size of TCGA dataset we used for the prognostic model creation was limited to 84 patients. Third, present study was retrospective validation with small sample sizes. Therefore, our conclusion should be further validated by prospective study in multicentre clinical trials. Fourth, our analysis is limited to comparison of tumor and normal only. MiRNAs that distinguish invasive *vs*. non-invasive BCa and different BCa histological subtype were also needed to explore. Lastly, current miRNA profiles are only limited to tissue. From the clinical point of view, it would be more interesting to explore non-invasive biomarkers associated with patient diagnosis, survival, disease aggressiveness or response to therapy. For this tissue resection based miRNA classfier would be just suitable for BCa prediction after cystectomy. Considering on this, we are now exploring non-invasive circulating miRNAs in plasma and urine which would be more meaningful for early detection and precise prediction.

## Conclusions

In summary, we identified a meta-signature, consisting of eight highly significant and consistently dysregulated miRNAs (miR-141, miR-21, miR-200c, miR-145, miR-125b, miR-199a, let-7c and miR-99a) from 19 different datasets. Using TCGA dataset, we constructed a prognostic model of eight-miRNA signature, also our validation cohort confirmed that the panel may be a useful biomarker for prognosis in BCa. We acknowledge that prospective, large-scale, multicentre studies are necessary to confirm our results before this miRNA signature can be applied into clinical practice, and the tumorigenic mechanisms of these miRNAs in BCa warrants further investigated.

## Additional files

Additional file 1: Figure S2. Flow chart of datasets identified, included, and excluded. Additional file 2: Figure S1. Overview of the design strategy. Additional file 3: Table S1. Characteristics of analyzed datasets. Additional file 4: Table S2. Up-regulated miRNAs (n=54) reported in at least three expression profiling studies. Additional file 5: Table S3. Down-regulated miRNAs (n=49) reported in at least three expression profiling studies. Additional file 6: Table S4. miRNAs dysregulated in bladder cancer and their involvement in cancer pathogenesis in other malignancies. Additional file 7: Figure S12. ROC curves for the 8-miRNA signature in TCGA cohort. The ROC curve had an AUC of 0.74 (*p* < 0.01). Additional file 8: Figure S3. Prognostic index and K-M analysis for miR-141 expression in patients with bladder cancer overall survival in TCGA datasets. Information related to censoring event being analyzed (risk group assignment, censoring status, time related to event, and prognostic index). Additional file 9: Figure S4. Prognostic index and K-M analysis for miR-200c expression in patients with bladder cancer overall survival in TCGA datasets. Information related to censoring event being analyzed (risk group assignment, censoring status, time related to event, and prognostic index) (A). Each patient’ prognostic index (risk score) divided them into high risk and low risk two groups (B). Survival analysis showed significant difference between BCa overall survival and miRNA expression (C). Additional file 10: Figure S5. Prognostic index and K-M analysis for miR-21 expression in patients with bladder cancer overall survival in TCGA datasets. Information related to censoring event being analyzed (risk group assignment, censoring status, time related to event, and prognostic index) (A). Each patient’ prognostic index (risk score) divided them into high risk and low risk two groups (B). Survival analysis showed significant difference between BCa overall survival and miRNA expression (C). Additional file 11: Figure S6. Prognostic index and K-M analysis for miR-145 expression in patients with bladder cancer overall survival in TCGA datasets. Information related to censoring event being analyzed (risk group assignment, censoring status, time related to event, and prognostic index) (A). Each patient’ prognostic index (risk score) divided them into high risk and low risk two groups (B). Survival analysis showed significant difference between BCa overall survival and miRNA expression (C). Additional file 12: Figure S7. Prognostic index and K-M analysis for miR-125b expression in patients with bladder cancer overall survival in TCGA datasets. Information related to censoring event being analyzed (risk group assignment, censoring status, time related to event, and prognostic index) (A). Each patient’ prognostic index (risk score) divided them into high risk and low risk two groups (B). Survival analysis showed significant difference between BCa overall survival and miRNA expression (C). Additional file 13: Figure S8. Prognostic index and K-M analysis for miR-199a expression in patients with bladder cancer overall survival in TCGA datasets. Information related to censoring event being analyzed (risk group assignment, censoring status, time related to event, and prognostic index) (A). Each patient’ prognostic index (risk score) divided them into high risk and low risk two groups (B). Survival analysis showed significant difference between BCa overall survival and miRNA expression (C). Additional file 14: Figure S9. Prognostic index and K-M analysis for let-7c expression in patients with bladder cancer overall survival in TCGA datasets. Information related to censoring event being analyzed (risk group assignment, censoring status, time related to event, and prognostic index) (A). Each patient’ prognostic index (risk score) divided them into high risk and low risk two groups (B). Survival analysis showed significant difference between BCa overall survival and miRNA expression (C). Additional file 15: Figure S10. Prognostic index and K-M analysis for miR-99a expression in patients with bladder cancer overall survival in TCGA datasets. Information related to censoring event being analyzed (risk group assignment, censoring status, time related to event, and prognostic index) (A). Each patient’ prognostic index (risk score) divided them into high risk and low risk two groups (B). Survival analysis showed significant difference between BCa overall survival and miRNA expression (C). Additional file 16: Figure S11. Hazard ratio of relative miRNAs and bladder cancer overall survival in TCGA datasets. Additional file 17: Table S5. Estimation of the hazard ratio on relative miRNAs expression and bladder cancer overall survival in TCGA datasets. Additional file 18: Table S6. Patients’ clinicopathological characters in our validation cohort (N = 48). Additional file 19: Figure S13. Expression of the eight-miRNAs signature in our validation cohort of 48 bladder cancer patients. Relative miRNA fold change (log transformed) in bladder cancer compared with normal adjacent tissue determined by qRT-PCR (miR-21 (A), miR-200c (B), miR-141 (C), miR-145 (D), miR-125b (E), miR-199a (F), miR-99a(G), let-7c (H)).
