# Supplementary figures and images for: A panel of eight-miRNA signature as a potential biomarker for predicting survival in bladder cancer
Source: J Exp Clin Cancer Res. 2015 May 21;34(1):53. doi: 10.1186/s13046-015-0167-0 (PMC4508815; doi:10.1186/s13046-015-0167-0)

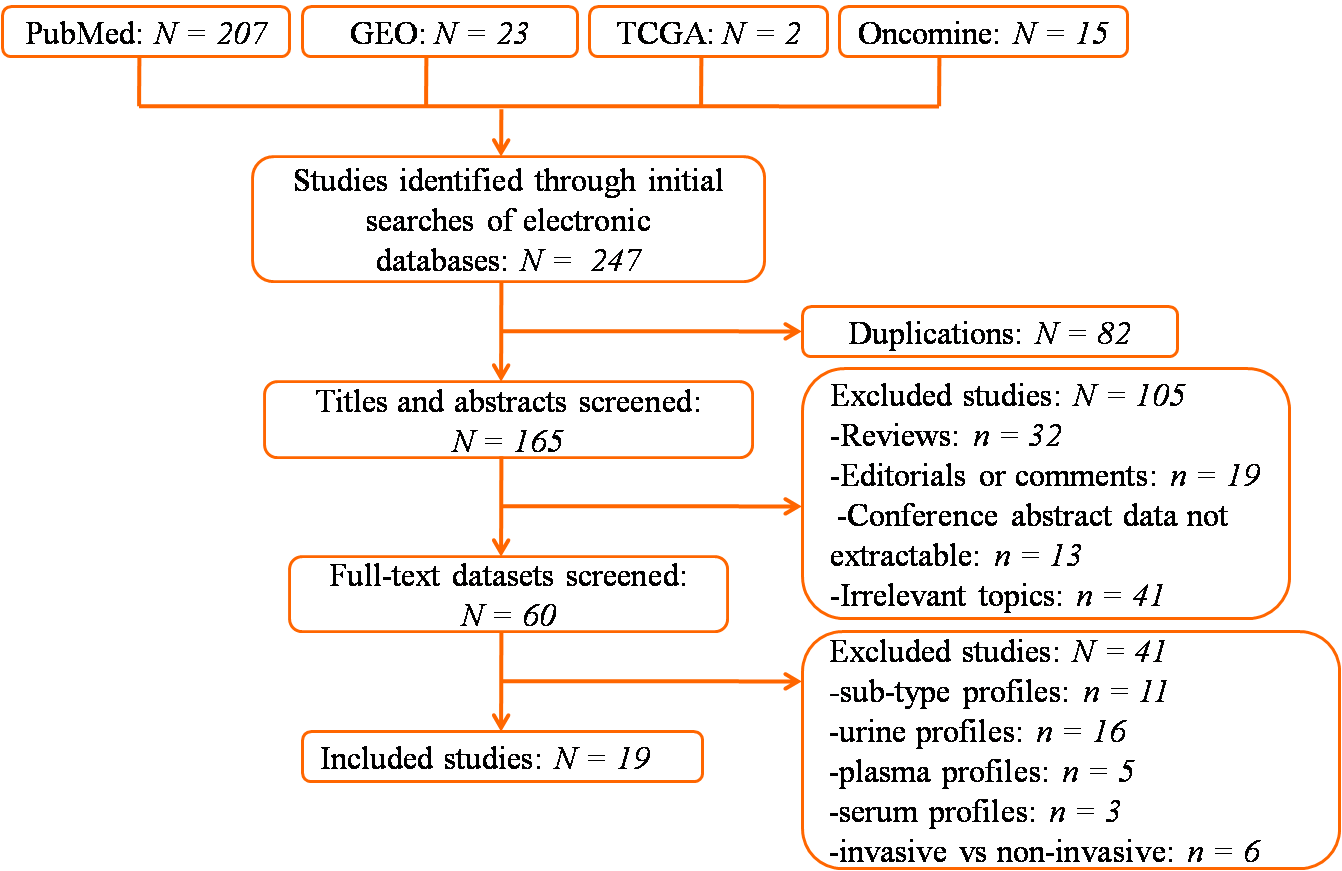

Supplement: Additional file 1: Figure S2. — Flow chart of datasets identified, included, and excluded. [file 13046_2015_167_MOESM1_ESM.tif]

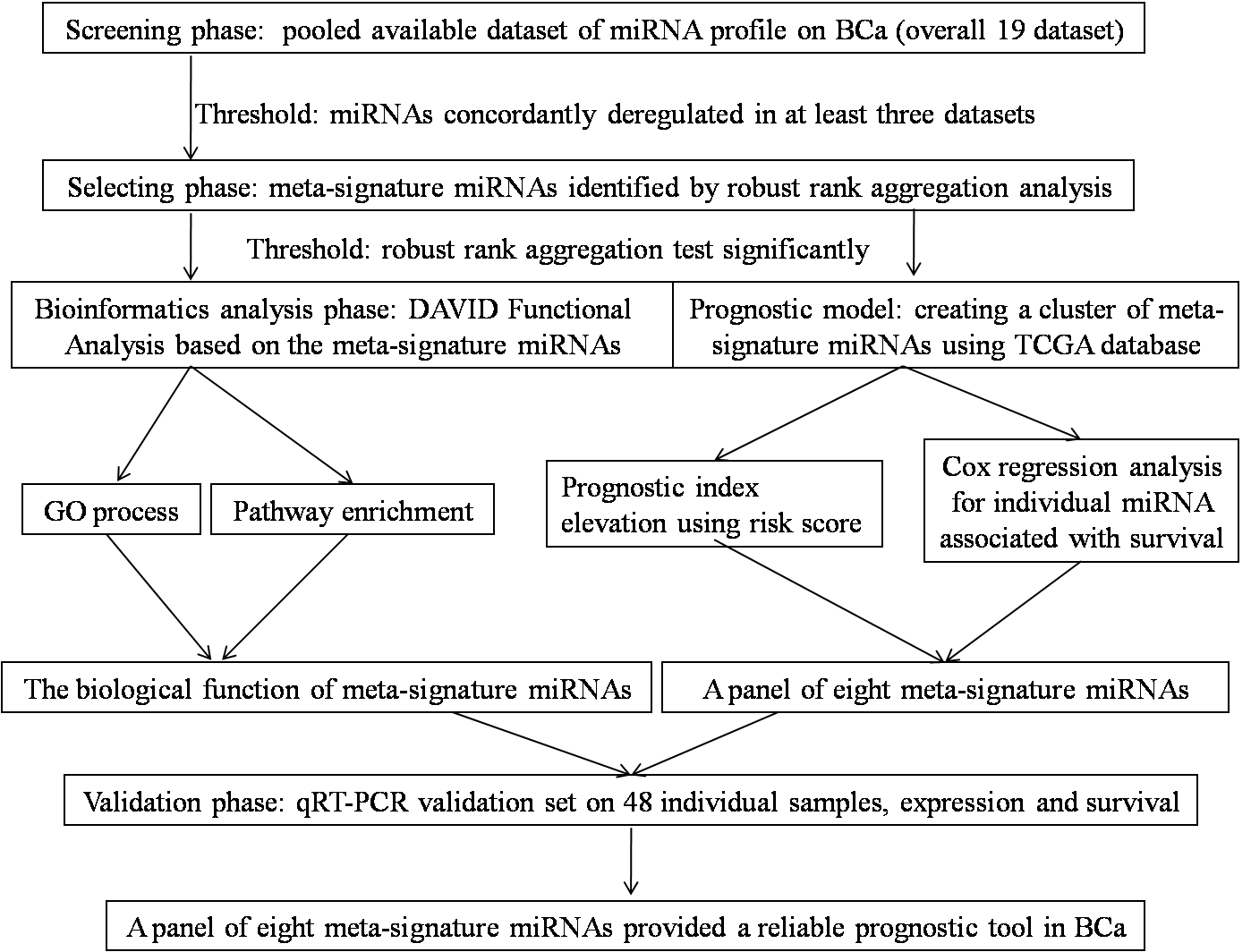

Supplement: Additional file 2: Figure S1. — Overview of the design strategy. [file 13046_2015_167_MOESM2_ESM.tif]

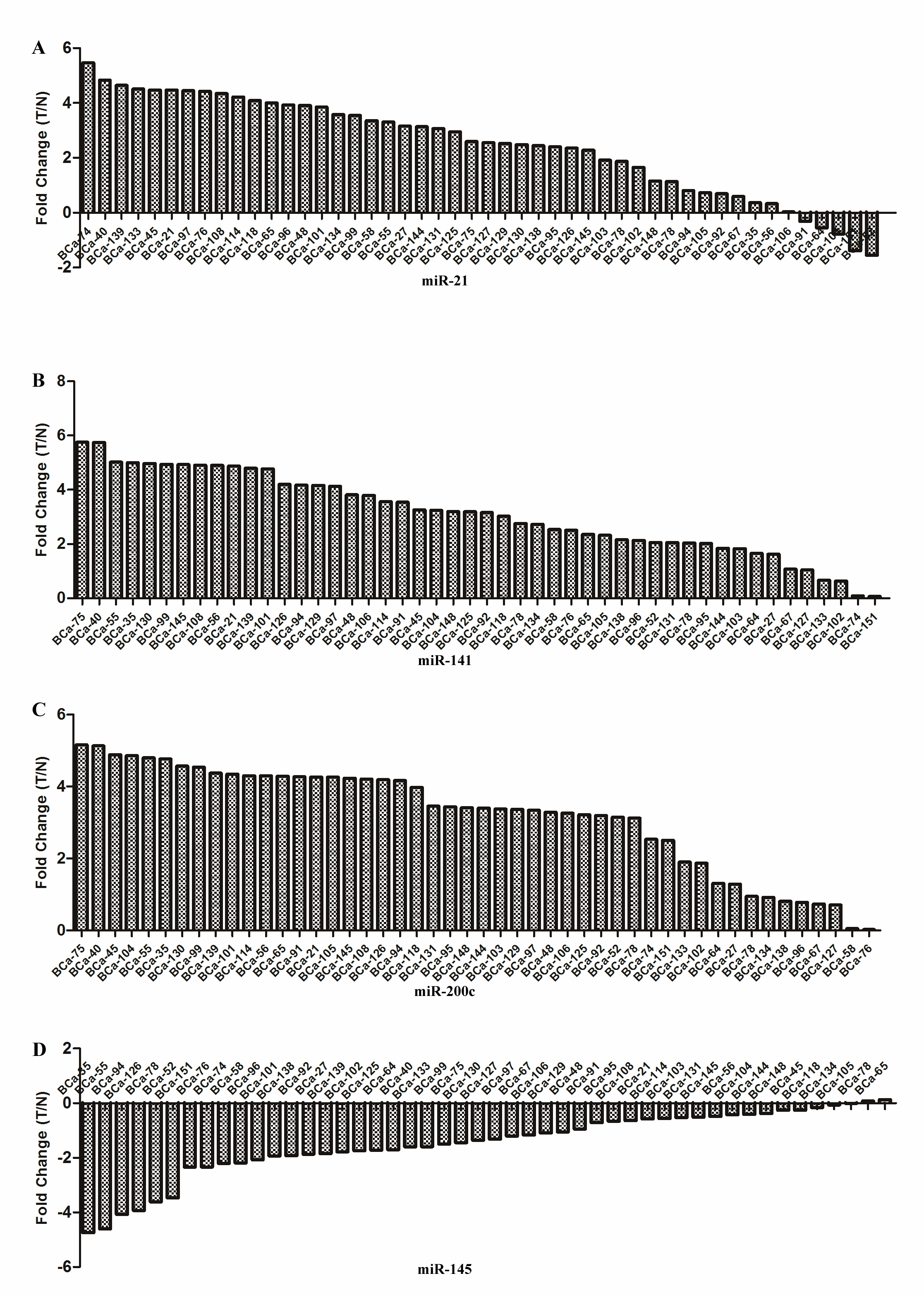

Supplement: Additional file 19: Figure S13. — Expression of the eight-miRNAs signature in our validation cohort of 48 bladder cancer patients. Relative miRNA fold change (log transformed) in bladder cancer compared with normal adjacent tissue determined by qRT-PCR (miR-21 (A), miR-200c (B), miR-141 (C), miR-145 (D), miR-125b (E), miR-199a (F), miR-99a(G), let-7c (H)). [file 13046_2015_167_MOESM19_ESM.zip › Fig. S13a-d.tif]

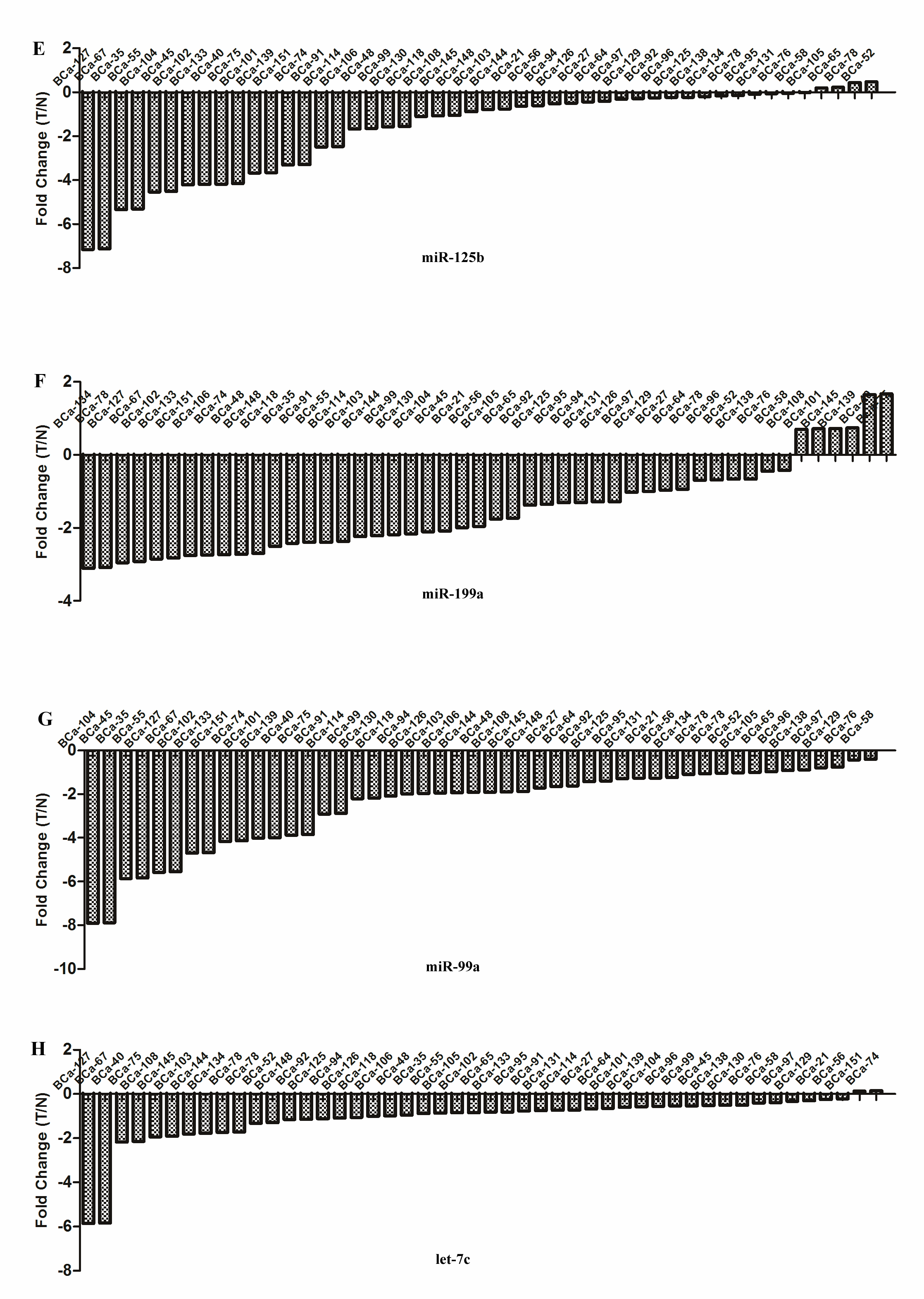

Supplement: Additional file 19: Figure S13. — Expression of the eight-miRNAs signature in our validation cohort of 48 bladder cancer patients. Relative miRNA fold change (log transformed) in bladder cancer compared with normal adjacent tissue determined by qRT-PCR (miR-21 (A), miR-200c (B), miR-141 (C), miR-145 (D), miR-125b (E), miR-199a (F), miR-99a(G), let-7c (H)). [file 13046_2015_167_MOESM19_ESM.zip › Fig. S13e-h.tif]
