# Supplementary material for: A panel of eight-miRNA signature as a potential biomarker for predicting survival in bladder cancer
Source: J Exp Clin Cancer Res. 2015 May 21;34(1):53. doi: 10.1186/s13046-015-0167-0 (PMC4508815; doi:10.1186/s13046-015-0167-0)
Supplement: Additional file 3: Table S1. — Characteristics of analyzed datasets. [file 13046_2015_167_MOESM3_ESM.doc]

**Table S1** Characteristics of analyzed datasets.

| Reference | Acronym | Country | Period | Cancer type | Clinical stage | No. of sample (T/N) | Microarray platform | No. of probes | Cut-off criteria |
| --- | --- | --- | --- | --- | --- | --- | --- | --- | --- |
| Catto,2009 | CA | UK | NA | UCC;NBE | pTa-T4 | 72(52/20) | 2100 Bioanalyzer (Agilent, Cheshire) | 322 | P < 0.05 and FDR 0.01; |
| Chen,2013 | CH | China | 2008-2010 | BCa, NBE | Ta-T4a | 30(20/10) | Illumina 1G genome analyzer(Illumina) | 884 | 1-FC (log2 ratio), p<0.001 |
| Dyrskjøt,2009 | DY | Denmark | NA | BCa, NBE | Ta-T4 | 117(106/11) | oligonucleotide probe library (Mercury LNA array,Exiqon) | 290 | P < 0.0001 |
| Friedman,2009 | FR | USA | NA | BCa,NBE | NA | 18(9/9) | miRNA microarray chip (LC science) | 313 | NA |
| Gottardo,2007 | GO | USA, Italy | 1998-2000 | UCC,NBE | Ta-T4 | 40(20/20) | KCC/TJU micro-RNA oligonucleotide microchips | 368 | 1.2-FC (log2 ratio), P<0.05 |
| Han,2011 | HA | China | NA | BCa, NBE | T1,T2,T4 | 18(9/9) | Illumina Cluster Station and Genome Analyze (Illumina) | 656 | FDR≤0.1%, p<0.01 |
| Ichimi,2009 | IC | Japan | 2003-2007 | BCa, NBE | pTa-T4 | 28(14/14) | GeneSpring GX version 7.3.1 software (Agilent Technologies) | 156 | FC >2.0 or <0.5 |
| Itesako,2014 | IT | Japan | 2003-2010 | BCa, NBE | T2-T3 | 10(5/5) | Genome Analyzer IIx (GAIIx) (Illumina) | 950 | FDR<0.01 |
| Li,2011 | LI | China | NA | TCC, NBE | NA | 20(10/10) | Illumina GAII platform (Illumina Inc, USA) | 226 | log2 ratio ≥1, FDR≤0.1 |
| Lin,2009 | LN | China | NA | BCa, NBE | T1-T4 | 12(6/6) | CapitalBio (Capital-Bio Corp., Beijing, China) | 509 | absolute value of FC≥2 |
| Pignot,2013 | PI | France | 2001-2005 | BCa, NBE | pTa-T4 | 28(14/14) | Human miScript Primer Assays(Qiagen) | 804 | P≤0.05 |
| Ratert,2013 | RA | Germany | 1998-2009 | BCa, NBE | PTa-T2 | 24(16/8) | human catalog 8-plex 15K miRNA microarrays (Agilent) | 723 | FDR-adjusted, P value≤0.05 |
| Song,2010 | SO | China | NA | BCa, NBE | NA | 50(25/25) | LC-miRNA microarray (μParaflo™, LC Sciences) | 51 | FC >2.0, p≤0.05 |
| Wiklund,2010 | WI | Denmark | NA | BCa, NBE | Ta-T4 | 68(57/11) | miRcury LNA array (Exiqon) | NA | NA |
| Xu,2013 | XU | China | 2008-2010 | BCa, NBE | Ta-T4 | 20(10/10) | 20 GeneChip miRNA Array (Affymetrix) | 6703 | RVM t test: value of ≥2 or ≤0.5 |
| Yoshino,2011 | YO | Japan | 2006-2009 | BCa, NBE | NA | 16(11/5) | TaqMan LDA Human microRNA Panel v2.0 (Biosystems) | 60 | P<0.05 |
| Zaravinos,2012 | ZA | Greece | NA | BCa, NBE | NA | 154(77/77) | miScript SYBR® Green PCR Kit | 11 | P<0.05 |
| Zhao,2013 | ZH | China | NA | BCa, NBE | NA | 22(14/8) | CFX96™ Real-Time PCR Detection System (Bio-Rad, CA) | 20 | P<0.05 |
| Zhu,2011 | ZU | China | NA | BCa, NBE | NA | 18(9/9) | Agilent 2100 Bioanalyzer (Agilent, US) | 196 | FDR P-value ≤0.05 |

BCa, bladder cancer; NBE, normal bladder epithelia; TCC, Transitional cell carcinoma ; UCC, Urothelial cell carcinoma; qRT-PCR, quantitative real-time polymerase chain reaction; FC, fold change; FDR, false discovery rate; NA, data not available.

**References**

1. Catto JW, Miah S, Owen HC, Bryant H, Myers K, Dudziec E, et al. Distinct microRNA alterations characterize high- and low-grade bladder cancer**.** Cancer Res. 2009; 69**:**8472-81.

2. Chen YH, Wang SQ, Wu XL, Shen M, Chen ZG, Chen XG, et al. Characterization of microRNAs expression profiling in one group of Chinese urothelial cell carcinoma identified by Solexa sequencing**.** Urol Oncol. 2013; 31**:**219-27.

3. Dyrskjot L, Ostenfeld MS, Bramsen JB, Silahtaroglu AN, Lamy P, Ramanathan R, et al. Genomic profiling of microRNAs in bladder cancer: miR-129 is associated with poor outcome and promotes cell death in vitro**.** Cancer Res. 2009; 69**:**4851-60.

4. Friedman JM, Liang G, Liu CC, Wolff EM, Tsai YC, Ye W, et al. The putative tumor suppressor microRNA-101 modulates the cancer epigenome by repressing the polycomb group protein EZH2**.** Cancer Res. 2009; 69**:**2623-9.

5. Gottardo F, Liu CG, Ferracin M, Calin GA, Fassan M, Bassi P, et al. Micro-RNA profiling in kidney and bladder cancers**.** Urol Oncol. 2007; 25**:**387-92.

6. Han Y, Chen J, Zhao X, Liang C, Wang Y, Sun L, et al. MicroRNA expression signatures of bladder cancer revealed by deep sequencing**.** PLoS One. 2011; 6**:**e18286.

7. Ichimi T, Enokida H, Okuno Y, Kunimoto R, Chiyomaru T, Kawamoto K, et al. Identification of novel microRNA targets based on microRNA signatures in bladder cancer**.** Int J Cancer. 2009; 125**:**345-52.

8. Itesako T, Seki N, Yoshino H, Chiyomaru T, Yamasaki T, Hidaka H, et al. The microRNA expression signature of bladder cancer by deep sequencing: the functional significance of the miR-195/497 cluster**.** PLoS One. 2014; 9**:**e84311.

9. Li X, Chen J, Hu X, Huang Y, Li Z, Zhou L, et al. Comparative mRNA and microRNA expression profiling of three genitourinary cancers reveals common hallmarks and cancer-specific molecular events**.** PLoS One. 2011; 6**:**e22570.

10. Lin T, Dong W, Huang J, Pan Q, Fan X, Zhang C, et al. MicroRNA-143 as a tumor suppressor for bladder cancer**.** J Urol. 2009; 181**:**1372-80.

11. Pignot G, Cizeron-Clairac G, Vacher S, Susini A, Tozlu S, Vieillefond A, et al. microRNA expression profile in a large series of bladder tumors: identification of a 3-miRNA signature associated with aggressiveness of muscle-invasive bladder cancer**.** Int J Cancer. 2013; 132**:**2479-91.

12. Ratert N, Meyer HA, Jung M, Lioudmer P, Mollenkopf HJ, Wagner I, et al. miRNA profiling identifies candidate mirnas for bladder cancer diagnosis and clinical outcome**.** J Mol Diagn. 2013; 15**:**695-705.

13. Song T, Xia W, Shao N, Zhang X, Wang C, Wu Y, et al. Differential miRNA expression profiles in bladder urothelial carcinomas**.** Asian Pac J Cancer Prev. 2010; 11**:**905-11.

14. Wiklund ED, Bramsen JB, Hulf T, Dyrskjot L, Ramanathan R, Hansen TB, et al. Coordinated epigenetic repression of the miR-200 family and miR-205 in invasive bladder cancer**.** Int J Cancer. 2011; 128**:**1327-34.

15. Xu C, Zeng Q, Xu W, Jiao L, Chen Y, Zhang Z, et al. miRNA-100 inhibits human bladder urothelial carcinogenesis by directly targeting mTOR**.** Mol Cancer Ther. 2013; 12**:**207-19.

16. Yoshino H, Chiyomaru T, Enokida H, Kawakami K, Tatarano S, Nishiyama K, et al. The tumour-suppressive function of miR-1 and miR-133a targeting TAGLN2 in bladder cancer**.** Br J Cancer. 2011; 104**:**808-18.

17. Zaravinos A, Radojicic J, Lambrou GI, Volanis D, Delakas D, Stathopoulos EN, et al. Expression of miRNAs involved in angiogenesis, tumor cell proliferation, tumor suppressor inhibition, epithelial-mesenchymal transition and activation of metastasis in bladder cancer**.** J Urol. 2012; 188**:**615-23.

18. Zhao YG, Li Y, Wang L, Yang H, Wang QT, Qi HY, et al. microRNA response elements-regulated TRAIL expression shows specific survival-suppressing activity on bladder cancer**.** Journal of Experimental & Clinical Cancer Research. 2013; 32.

19. Zhu J, Jiang Z, Gao F, Hu X, Zhou L, Chen J, et al. A systematic analysis on DNA methylation and the expression of both mRNA and microRNA in bladder cancer**.** PLoS One. 2011; 6**:**e28223.
