# Supplementary material for: A panel of eight-miRNA signature as a potential biomarker for predicting survival in bladder cancer
Source: J Exp Clin Cancer Res. 2015 May 21;34(1):53. doi: 10.1186/s13046-015-0167-0 (PMC4508815; doi:10.1186/s13046-015-0167-0)
Supplement: Additional file 4: Table S2. — Up-regulated miRNAs (n=54) reported in at least three expression profiling studies. [file 13046_2015_167_MOESM4_ESM.doc]

**Table S2** Up-regulated miRNAs (n=54) reported in at least three expression profiling studies

| miRNA | Reference | No. | Fold change | | | | | | | | |
| --- | --- | --- | --- | --- | --- | --- | --- | --- | --- | --- | --- |
| 200a-3p | 2,6,9,10,12,15,13,14,19 | 9 | -2.30 | 6.87 | 8.30 | 14.90 | 22.10 | 4.66 | 1.43 | 3.50 | 5.26 |
| 205-5p | 2,5,9,12,13,14,17,18,19 | 9 | 8.00 | 4.05 | 7.86 | 9.71 | 11.96 | 2.49 | 2.41 | -6.67 | 3.93 |
| 182-5p | 2,4,6,8,9,11,13,15,19 | 9 | 7.46 | 37.00 | 17.00 | 3.43 | 18.41 | 68.60 | 14.90 | -2.30 | 14.00 |
| 141-3p | 1,2,6,9,12,13,14,19 | 8 | 14.35 | -6.80 | 8.22 | 9.72 | 27.20 | 4.32 | 1.92 | 5.84 |  |
| 200b-3p | 2,6,9,11,12,13,14,19 | 8 | 2.30 | 6.73 | 7.86 | 199.70 | 19.80 | 32.20 | 1.78 | 4.67 |  |
| 200c-3p | 2,6,9,10,12,13,14,19 | 8 | 3.48 | 6.87 | 7.82 | 13.80 | 16.30 | 18.60 | 1.99 | 4.61 |  |
| 210-5p | 9,10,11,12,13,15,17,19 | 8 | 10.97 | 19.60 | 1.90 | 8.95 | 5.46 | 3.33 | 8.74 | 12.64 |  |
| 20a-5p | 3,6,9,10,12,13,15,19 | 8 | 2.10 | 3.12 | 3.54 | 10.60 | 2.78 | 2.95 | 3.51 | 3.28 |  |
| 183-5p | 2,4,6,7,9,13,19 | 7 | 11.30 | 38.00 | 14.10 | 40.84 | 14.80 | 8.17 | 10.30 |  |  |
| 17-5p | 5,6,9,10,12,13,19 | 7 | 1.44 | 3.32 | 3.60 | 4.10 | 2.79 | 2.81 | 3.43 |  |  |
| 93-5p | 1,2,9,10,12,13,19 | 7 | 15.63 | 1.30 | 3.84 | 10.50 | 2.66 | 3.27 | 3.23 |  |  |
| 21-5p | 1,3,9,13,17,19 | 6 | 35.45 | 3.70 | 4.38 | 3.56 | 4.52 | 3.07 |  |  |  |
| 25-3p | 2,6,9,10,13,19 | 6 | 3.25 | 5.28 | 2.48 | 6.70 | 6.28 | 2.11 |  |  |  |
| 10a-5p | 1,2,4,9,13,19 | 6 | 16.92 | -2.46 | 10.00 | 4.89 | 10.50 | 6.23 |  |  |  |
| 106a-5p | 7,9,10,13,15,19 | 6 | 6.36 | 3.26 | 4.90 | 3.05 | 2.52 | 2.98 |  |  |  |
| 19a-3p | 6,9,10,12,17,19 | 6 | 4.15 | 4.63 | 9.90 | 5.11 | -2.10 | 4.75 |  |  |  |
| 429-5p | 6,9,12,13,14,19 | 6 | 8.40 | 27.20 | 9.84 | 11.70 | NC | 5.58 |  |  |  |
| 106b-5p | 2,9,12,13,15,19 | 6 | -2.83 | 4.28 | 3.84 | 2.69 | 3.56 | 3.80 |  |  |  |
| 181b-5p | 1,2,9,13,19 | 5 | 15.06 | 2.14 | 4.91 | 3.23 | 3.91 |  |  |  |  |
| let-7b | 1,2,9,13,19 | 5 | 46.59 | 2.70 | 2.26 | 1.25 | 2.30 |  |  |  |  |
| 224-5p | 2,4,7,9,19 | 5 | 9.85 | 23.00 | 28.12 | 5.65 | 5.50 |  |  |  |  |
| 146b-5p | 2,9,11,13,19 | 5 | 6.06 | 2.23 | 3.90 | 3.58 | 2.66 |  |  |  |  |
| 34a-5p | 1,9,12,15,19 | 5 | 25.51 | 4.74 | 2.15 | 3.03 | 3.75 |  |  |  |  |
| 185-5p | 5,9,13,15,19 | 5 | 1.30 | 4.42 | 2.06 | 2.15 | 3.87 |  |  |  |  |
| 181a-5p | 2,9,13,19 | 4 | 3.25 | 3.32 | 2.00 | 2.37 |  |  |  |  |  |
| 151-3p | 9,13,15,19 | 4 | 3.03 | 3.18 | 2.41 | 2.67 |  |  |  |  |  |
| 130b-3p | 7,9,12,19 | 4 | 2.84 | 5.35 | 3.71 | 4.29 |  |  |  |  |  |
| 19b-3p | 9,12,17,19 | 4 | 4.52 | 3.39 | -1.28 | 4.28 |  |  |  |  |  |
| 7-5p | 6,9,12,19 | 4 | 2.55 | 6.59 | 22.20 | 9.17 |  |  |  |  |  |
| 15a-5p | 1,9,13,19 | 4 | 15.21 | 4.08 | 3.94 | 3.55 |  |  |  |  |  |
| 92a-3p | 2,9,13,19 | 4 | 3.73 | 2.12 | 5.17 | 4.22 |  |  |  |  |  |
| 425-5p | 9,12,15,19 | 4 | 6.52 | 5.82 | 2.51 | 5.50 |  |  |  |  |  |
| 96-5p | 6,7,19 | 3 | 25.36 | 25.44 | 16.89 |  |  |  |  |  |  |
| 378-5p | 1,13,17 | 3 | 16.53 | 1.54 | -3.50 |  |  |  |  |  |  |
| 30e-5p | 9,13,19 | 3 | 2.49 | 2.62 | 2.41 |  |  |  |  |  |  |
| 148b-3p | 2,13,19 | 3 | -2.64 | 4.06 | 2.00 |  |  |  |  |  |  |
| 26b-5p | 2,5,13 | 3 | -4.29 | 1.31 | 2.16 |  |  |  |  |  |  |
| 18a-5p | 9,10,19 | 3 | 5.90 | 2.90 | 5.68 |  |  |  |  |  |  |
| 9-5p | 7,11,19 | 3 | -12.50 | 1.00 | 2.86 |  |  |  |  |  |  |
| 24-3p | 9,13,19 | 3 | 2.22 | -2.75 | 2.30 |  |  |  |  |  |  |
| 200b-5p | 2,9,13,19 | 3 | 5.27 | 9.18 | 5.21 |  |  |  |  |  |  |
| 203a-3p | 4,9,19 | 3 | 36.00 | 8.49 | 5.43 |  |  |  |  |  |  |
| 146a-5p | 9,13,19 | 3 | 5.30 | 7.01 | 4.34 |  |  |  |  |  |  |
| 423-5p | 2,9,13 | 3 | 4.00 | 2.41 | 2.69 |  |  |  |  |  |  |
| let-7a | 1,2,13 | 3 | 18.43 | 2.83 | -1.23 |  |  |  |  |  |  |
| let-7d | 1,2,13 | 3 | 20.92 | 2.14 | 1.21 |  |  |  |  |  |  |
| 16-5p | 9,13,19 | 3 | 2.21 | 1.49 | 3.32 |  |  |  |  |  |  |
| 345-5p | 1,9,19 | 3 | 26.62 | 3.77 | 3.03 |  |  |  |  |  |  |
| 452-5p | 1,9,19 | 3 | 13.41 | 3.11 | 2.86 |  |  |  |  |  |  |
| 181d-5p | 1,9,19 | 3 | 11.60 | 2.14 | 2.29 |  |  |  |  |  |  |
| 196a-5p | 2,4,19 | 3 | 45.25 | 23.00 | 4.59 |  |  |  |  |  |  |
| 30b-5p | 9,13,19 | 3 | 2.08 | 2.39 | 2.08 |  |  |  |  |  |  |
| 181a-3p | 9,15,19 | 3 | 3.59 | -2.30 | 2.10 |  |  |  |  |  |  |
| 181a-2-3p | 8,9,19 | 3 | -2.30 | 5.25 | 3.47 |  |  |  |  |  |  |

miRNA expression fold-change: down-regulated are in black and up-regulated are in red
