# Supplementary material for: A panel of eight-miRNA signature as a potential biomarker for predicting survival in bladder cancer
Source: J Exp Clin Cancer Res. 2015 May 21;34(1):53. doi: 10.1186/s13046-015-0167-0 (PMC4508815; doi:10.1186/s13046-015-0167-0)
Supplement: Additional file 5: Table S3. — Down-regulated miRNAs (n=49) reported in at least three expression profiling studies. [file 13046_2015_167_MOESM5_ESM.doc]

**Table S3** Down-regulated miRNAs (n=49) reported in at least three expression profiling studies

| miRNA | Reference | No. | Fold change | | | | | | | |
| --- | --- | --- | --- | --- | --- | --- | --- | --- | --- | --- |
| 145-5p | 2,3,4,6,7,8,9,10,11,12,13,15,16,17,18,19 | 16 | -26.00 | -7.00 | -2.82 | -8.88 | -33.30 | -49.73 | -8.68 | -15.90 |
|  |  |  | -174.90 | -46.60 | -657.00 | -2.50 | -15.67 | -2.60 | -5.26 | -4.71 |
| 143-3p | 2,3,4,6,8,9,10,11,12,13,15,16,18,19 | 14 | -42.40 | -2.60 | -11.00 | -7.62 | -19.49 | -6.49 | -13.70 | -138.80 |
|  |  |  | -46.40 | -56.10 | -2.33 | -4.46 | -2.94 | -5.13 |  |  |
| 125b-5p | 2,3,6,7,8,9,10,12,13,15,18,19 | 12 | -2.46 | -2.30 | -9.99 | -20.00 | -18.10 | -9.72 | -24.90 | -29.40 |
|  |  |  | -3.70 | -3.13 | -3.30 | -4.99 |  |  |  |  |
| 1-3p | 2,4,6,8,9,11,13,16,17,18,19 | 11 | -294.10 | -54.00 | -15.70 | -126.85 | -16.91 | -249.90 | -657.00 | -21.19 |
|  |  |  | -10.30 | -21.20 | -11.10 |  |  |  |  |  |
| 195-5p | 6,7,8,9,10,12,13,16,18,19 | 10 | -4.32 | -3.85 | -9.30 | 2.63 | -8.10 | -6.69 | -8.82 | -3.18 |
|  |  |  | -5.56 | -2.97 |  |  |  |  |  |  |
| 100-5p | 2,6,7,8,9,12,13,15,18,19 | 10 | -59.70 | 5.17 | -20.00 | -12.74 | -5.64 | -80.00 | -44.30 | -2.84 |
|  |  |  | -2.32 | -3.25 |  |  |  |  |  |  |
| 133a-5p | 6,7,8,9,11,13,16,18,19 | 9 | -18.40 | -50.00 | -110.35 | -19.27 | -183.80 | -33.10 | -47.17 | -16.70 |
|  |  |  | -8.67 |  |  |  |  |  |  |  |
| let-7c | 1,2,6,8,9,12,16,18,19 | 9 | 20.35 | 5.28 | -2.52 | -39.81 | -5.77 | -2.06 | -6.58 | -5.26 |
|  |  |  | -3.42 |  |  |  |  |  |  |  |
| 99a-5p | 6,7,8,9,13,15,18,19 | 8 | -16.10 | -16.70 | -25.16 | -14.08 | -28.10 | -4.90 | -12.50 | -4.55 |
| 199a-3p | 7,8,9,11,12,13,18,19 | 8 | -9.09 | -4.37 | -3.30 | -12.10 | -7.07 | -16.70 | -2.32 | -2.52 |
| 139-5p | 7,8,9,12,13,16,19 | 7 | -14.30 | -23.13 | -6.54 | -364.00 | -4.47 | -17.86 | -3.24 |  |
| 152-5p | 2,7,8,9,13,16,19 | 7 | -3.25 | -5.56 | -3.44 | -2.43 | -3.58 | -2.17 | -2.03 |  |
| 214-3p | 9,12,13,15,16,19 | 6 | -2.50 | -5.01 | -34.90 | -20.25 | -6.99 | -2.83 |  |  |
| 133b | 1,7,8,9,16,19 | 6 | -28.25 | -33.30 | -158.79 | -16.54 | -14.16 | -12.71 |  |  |
| 23b-3p | 2,5,8,10,12,13 | 6 | -2.00 | 1.62 | -5.72 | -2.90 | -4.00 | -1.74 |  |  |
| 199a-5p | 8,9,12,13,16,19 | 6 | -3.82 | -3.48 | -9.73 | -15.24 | -3.36 | -2.79 |  |  |
| 126-3p | 2,3,10,16,17 | 5 | -3.70 | -2.00 | -3.60 | -3.39 | -2.10 |  |  |  |
| 204-5p | 1,8,9,11,16 | 5 | -27.47 | -52.06 | -3.38 | -66.00 | -21.69 |  |  |  |
| 223-3p | 5,7,8,12,19 | 5 | 1.49 | -11.10 | -6.83 | -3.74 | 2.33 |  |  |  |
| 145-3p | 6,8,9,12,19 | 5 | -6.89 | -21.26 | -7.04 | -674.00 | -4.84 |  |  |  |
| 490-5p | 8,9,12,18,19 | 5 | -32.27 | -37.22 | -148.00 | -21.15 | -11.94 |  |  |  |
| 199b-5p | 8,9,12,16,19 | 5 | -5.91 | -3.71 | -23.70 | -7.35 | -2.75 |  |  |  |
| 221-3p | 1,5,10,13,17 | 5 | 14.86 | 1.50 | -5.80 | -4.53 | -3.30 |  |  |  |
| 26a-5p | 2,3,12,13 | 4 | -3.48 | -1.60 | -2.49 | -1.25 |  |  |  |  |
| 101-3p | 2,4,18,19 | 4 | -6.00 | -16.00 | -10.80 | -2.14 |  |  |  |  |
| 29a-3p | 3,10,12,13 | 4 | -1.70 | -3.40 | -2.99 | -3.05 |  |  |  |  |
| 193a-5p | 2,10,13,19 | 4 | 6.96 | -8.80 | -3.18 | -2.79 |  |  |  |  |
| 22-3p | 2,12,13,15 | 4 | -9.51 | -2.24 | -3.18 | 2.16 |  |  |  |  |
| 130a-3p | 2,8,12,13 | 4 | -4.29 | -3.87 | -2.59 | -4.78 |  |  |  |  |
| 30a-5p | 7,8,12,13 | 4 | -25.00 | -4.59 | -5.22 | 1.43 |  |  |  |  |
| 497-5p | 8,9,12,19 | 4 | -6.20 | -2.98 | -7.20 | -2.76 |  |  |  |  |
| 3835p | 8,9,16,19 | 4 | -24.93 | -4.76 | -46.73 | -2.33 |  |  |  |  |
| 139-3p | 8,9,16,19 | 4 | -10.15 | -3.82 | -55.87 | -3.79 |  |  |  |  |
| 99a-3p | 8,9,16,19 | 4 | -41.04 | -24.35 | -2.75 | -3.81 |  |  |  |  |
| 218-5p | 7,16,18 | 3 | -7.10 | -4.10 | -12.50 |  |  |  |  |  |
| 29c-3p | 3,4,12 | 3 | -2.00 | -9.00 | 1.60 |  |  |  |  |  |
| 150-5p | 7,10,13 | 3 | -6.67 | -5.00 | 5.65 |  |  |  |  |  |
| 222-3p | 12,13,19 | 3 | -12.90 | -5.13 | 2.00 |  |  |  |  |  |
| 486-5p | 8,12,13 | 3 | -11.07 | -7.86 | 3.25 |  |  |  |  |  |
| 125b-1-3p | 8,9,19 | 3 | -7.20 | -4.03 | -2.45 |  |  |  |  |  |
| 125b-2-3p | 8,9,19 | 3 | -12.31 | -20.41 | -4.50 |  |  |  |  |  |
| 127-3p | 8,9,19 | 3 | -3.62 | -2.52 | -2.06 |  |  |  |  |  |
| 143-5p | 8,9,19 | 3 | -7.22 | -7.24 | -5.84 |  |  |  |  |  |
| 199b-3p | 8,9,19 | 3 | -4.37 | -3.30 | -2.52 |  |  |  |  |  |
| 30a-3p | 8,9,19 | 3 | -6.74 | -2.77 | -2.36 |  |  |  |  |  |
| 30c-2-3p | 8,9,16 | 3 | -6.06 | -2.25 | -1.81 |  |  |  |  |  |
| 338-3p | 8,9,19 | 3 | -4.44 | -2.26 | -2.25 |  |  |  |  |  |
| 338-5p | 8,9,19 | 3 | -9.61 | -2.76 | -2.25 |  |  |  |  |  |
| 490-3p | 8,9,19 | 3 | -69.70 | -25.84 | -5.63 |  |  |  |  |  |

miRNA expression fold-change: down-regulated are in black and up-regulated are in red
