# Supplementary material for: A panel of eight-miRNA signature as a potential biomarker for predicting survival in bladder cancer
Source: J Exp Clin Cancer Res. 2015 May 21;34(1):53. doi: 10.1186/s13046-015-0167-0 (PMC4508815; doi:10.1186/s13046-015-0167-0)
Supplement: Additional file 6: Table S4. — miRNAs dysregulated in bladder cancer and their involvement in cancer pathogenesis in other malignancies. [file 13046_2015_167_MOESM6_ESM.doc]

**Table S4** miRNAs dysregulated in bladder cancer and their involvement in cancer pathogenesis in other malignancies.

| miRNA | Cancer | Function | Target |
| --- | --- | --- | --- |
| miR-21 | Multiple cancers | Cellular growth, migration, invasion | PDCD4, RECK, TIMP3, MARKS, LRRF1P1, PTEN, SPROUTY2, TPM1, RHOB |
| miR-141 | Increased upon hypoxia | Increased cell survival and migration | Ephrin-A |
|  | Ovarian cancer | Regulate cell cycle | E2F3 |
|  | Multiple cancers | Response to hypoxia | MNT |
| miR-200c | HCC | Cell cycle and apoptosis | BCL-W, CCNG1, Cyclin G1 |
| miR-145 | Breast cancer | Tight junction dissolution, cell polarity, EMT | RHOA1 |
|  |  | Cell survival and chemoresistance | FOXO3 |
|  |  | Cell proliferation | SOCS1 |
|  | B-cell lymphoma | Cell proliferation | SHIP1 |
| miR-125b | HCC | Apoptotic cell death | API-5 |
| miR-199a | NSCLC | Cell proliferation | GPR124, PDK1 |
|  | HNSCC | Cellular death and invasion | RhoC |
|  | colorectal cancer | Metatstasis | TWIST2 |
| miR-99a | Multiple cancers | cell cycle arrest, DNA damage response, invasion and metastasis | mTOR,FGFR3,RAVER2,IGF1R |
| Let-7c | Multiple cancers | Cellular migration, apoptosis | HOXA1, TGFβR1,MMP11,PBX3,Bcl-xL |
| HCC, hepatoma cell carcinoma; NSCLC, nonsmall-cell lung cancer; HNSCC, head and neck squamous cell carcinoma. hsa-miR-200 family includes hsa-miR-200a, hsa-miR-200b, hsa-miR-200c, hsa-miR-141, and hsa-miR-429. | | | |
