# Supplementary material for: A panel of eight-miRNA signature as a potential biomarker for predicting survival in bladder cancer
Source: J Exp Clin Cancer Res. 2015 May 21;34(1):53. doi: 10.1186/s13046-015-0167-0 (PMC4508815; doi:10.1186/s13046-015-0167-0)
Supplement: Additional file 17: Table S5. — Estimation of the hazard ratio on relative miRNAs expression and bladder cancer overall survival in TCGA datasets. [file 13046_2015_167_MOESM17_ESM.doc]

**Table** **S5** Estimation of the hazard ratio on relative miRNAs expression and bladder cancer overall survival in TCGA datasets.

| **miRNA** | **HR (95% CI)** | ***p-*value** | **High risk** | **Low risk** |
| --- | --- | --- | --- | --- |
| miR-145-5p | 3.53 (1.28-9.68) | 0.01441 | 54 | 30 |
| miR-143-3p | 1.15 (0.50-2.67) | 0.7367 | 32 | 52 |
| miR-125b-5p | 4.83 (1.96-11.88) | 0.0006112 | 34 | 50 |
| miR-1-3p | 2.09 (0.62-7.06) | 0.2348 | 67 | 17 |
| miR-195-5p | 2.96 (1.15-7.64) | 0.02468 | 51 | 33 |
| miR-100-5p | 2.26 (0.93-5.47) | 0.07201 | 51 | 33 |
| miR-133a-5p | 0.55 (0.16-1.88) | 0.3435 | 17 | 67 |
| miR-99a-5p | 4.52 (1.83-11.16) | 0.0001072 | 37 | 47 |
| let-7c | 4.43 (1.85-10.59) | 0.000833 | 31 | 53 |
| miR-20a-5p | 2.65 (1.04-6.75) | 0.04117 | 49 | 35 |
| miR-199a-3p | 5.91 (1.95-17.93) | 0.001684 | 12 | 72 |
| miR-17-5p | 2.53 (1.09-5.86) | 0.03036 | 26 | 58 |
| miR-139-5p | 4.61 (1.25-16.96) | 0.02166 | 8 | 76 |
| miR-152-5p | 4.50 (1.89-10.67) | 0.0006519 | 22 | 62 |
| miR-214-3p | 2.57 (1.08-6.14) | 0.03334 | 20 | 64 |
| miR-133b | 2.01 (0.80-5.08) | 0.1383 | 12 | 72 |
| miR-23b-3p | 6.04 (0.81-44.87) | 0.07885 | 69 | 15 |
| miR-199a-5p | na | na | na | na |
| miR-29c-3p | 3.59 (1.50-8.58) | 0.004085 | 18 | 66 |
| miR-26a-5p | 0.30 (0.04-2.22) | 0.2378 | 8 | 76 |
| miR-221-3p | MAX | 0.9979 | 75 | 9 |
| miR-101-3p | 2.09 (0.92-4.77) | 0.07965 | 32 | 52 |
| miR-126-3p | 1.77 (0.79-3.99) | 0.1663 | 34 | 50 |
| miR-204-5p | 11.21 (1.51-88.33) | 0.0182 | 59 | 25 |
| miR-223-3p | 2.02 (0.74-5.55) | 0.1706 | 13 | 71 |
| miR-145-3p | na | na | na | na |
| miR-490-5p | 5.76 (0.77-43.09) | 0.08787 | 76 | 8 |
| miR-199b-5p | 2.39 (0.81-7.02) | 0.1145 | 10 | 74 |
| miR-378a-5p | 3.40 (1.16-9.98) | 0.02574 | 51 | 33 |
| miR-29a-3p | 3.25 (0.97-10.96) | 0.05705 | 61 | 23 |
| miR-200a-3p | 2.63 (1.11-6.22) | 0.02733 | 43 | 41 |
| miR-141-3p | 3.06 (1.34-7.00) | 0.008143 | 24 | 60 |
| miR-205-5p | 1.61 (0.70-3.68) | 0.2612 | 26 | 58 |
| miR-182-5p | 1.84 (0.80-4.24) | 0.1506 | 24 | 60 |
| miR-200b-3p | 3.96 (1.67-9.42) | 0.001817 | 31 | 53 |
| miR-200c-3p | 2.50 (1.06-5.88) | 0.03589 | 36 | 48 |
| miR-210-5p | 3.61 (1.07-12.19) | 0.0389 | 60 | 24 |
| miR-183-5p | 1.39 (0.59-3.25) | 0.4485 | 23 | 61 |
| miR-21-5p | 2.15 (0.88-5.25) | 0.09158 | 49 | 35 |
| miR-93-5p | 1.83 (0.81-4.13) | 0.1454 | 29 | 55 |
| miR-25-3p | 3.13 (1.19-8.24) | 0.0207 | 59 | 25 |
| miR-10a-5p | 2.39 (1.02-5.59) | 0.0442 | 26 | 58 |
| miR-106a-5p | 2.15 (0.87-5.30) | 0.09629 | 55 | 29 |
| miR-19a-3p | 3.19 (1.25-8.11) | 0.01487 | 49 | 35 |
| miR-429 | 8.59 (2.81-26.27) | 0.0001626 | 9 | 75 |
| miR-181a-5p | 2.85 (0.95-8.53) | 0.06048 | 10 | 74 |
| miR-181b-5p | 1.80 (0.81-4.04) | 0.1517 | 39 | 45 |
| miR-106b-5p | MAX | 0.9971 | 73 | 11 |
| let-7b | 0 | 0.9978 | 6 | 78 |
| miR-224-5p | 2.81 (1.03-7.67) | 0.04337 | 64 | 20 |
| miR-146b-5p | 0.53 (0.23-1.21) | 0.1304 | 37 | 47 |
| miR-151-3p | 2.87 (1.19-6.94) | 0.01901 | 20 | 64 |
| miR-34a-5p | 2.42 (1.03-5.68) | 0.04247 | 36 | 48 |
| miR-185-5p | 4.17 (1.54-11.3) | 0.005085 | 9 | 75 |
| miR-130b-3p | 3.79 (1.08-13.26) | 0.03727 | 69 | 15 |
| miR-30e-5p | 2.02 (0.86-4.76) | 0.1067 | 25 | 59 |
| miR-19b-3p | 7.61 (1.02-56.85) | 0.04799 | 68 | 16 |
| na, data not available. | | | | |
