# Supplementary material for: A panel of eight-miRNA signature as a potential biomarker for predicting survival in bladder cancer
Source: J Exp Clin Cancer Res. 2015 May 21;34(1):53. doi: 10.1186/s13046-015-0167-0 (PMC4508815; doi:10.1186/s13046-015-0167-0)
Supplement: Additional file 18: Table S6. — Patients’ clinicopathological characters in our validation cohort (N = 48). [file 13046_2015_167_MOESM18_ESM.doc]

**Table S6** Patients’ clinicopathological characters in our validation cohort (N = 48).

| Category | Value |
| --- | --- |
| No. patients | 48 |
| Age, median (range), yr | 56 (45-80) |
| BMI, mean ± SD, kg/m^2^ | 24.45 ± 2.68 |
| Male, No. (%) | 28 (58.33%) |
| Tumor multiplicity | 15 (31.25%) |
| Pathological stage, No. (%) |  |
| pTa | 13 (28.89) |
| pT1 | 24 (53.33) |
| pT2 | 4 (8.89) |
| pT3 | 2 (4.44) |
| pT4 | 1 (2.22) |
| Grade |  |
| G1 | 14 (31.11) |
| G2 | 28 (62.22) |
| G3 | 5 (6.67) |
| Local recurrence, No. (%) | 2 (4.44) |
| Metastasis, No. (%) | 1 (2.22) |
| BMI, body mass index; pT, pathological stage; G, grade. | |
